# Electroacupuncture attenuates inflammatory pain via peripheral cannabinoid receptor type 1 signaling pathway in mice

**DOI:** 10.1371/journal.pone.0295432

**Published:** 2023-12-07

**Authors:** Tsung-Jung Ho, Ching-Fang Lin, Jhong-Kuei Chen, Yen-Lun Kung, Li-Kung Wu, Chen-Ying Chang Chien, Chun-Ping Huang

**Affiliations:** 1 Integration Center of Traditional Chinese and Modern Medicine, Hualien Tzu Chi Hospital, Hualien, Taiwan; 2 Department of Chinese Medicine, Hualien Tzu Chi Hospital, Hualien, Taiwan; 3 School of Post‑Baccalaureate Chinese Medicine, Tzu Chi University, Hualien, Taiwan; 4 Institute of Medical Sciences, Tzu Chi University, Hualien, Taiwan; University of Turin, ITALY

## Abstract

Pain is strongly associated with neuro-immune activation. Thus, the emerging role of the endocannabinoid system in neuro-inflammation is important. Acupuncture has been used for over 2500 years and is widely accepted for the management of pain. Our study aimed to investigate the effects of electroacupuncture on the regulation of cannabinoid receptor type 1 within the peripheral nervous system. Inflammatory pain was induced by injecting Complete Freund’s adjuvant to induce mechanical and thermal hyperalgesia. Electroacupuncture significantly attenuated the mechanical and thermal sensitivities, and AM251, a cannabinoid receptor type 1 antagonist, eliminated these effects. Dual immunofluorescence staining demonstrated that electroacupuncture elevated expression of cannabinoid receptor type 1, co-localized with Nav 1.8. Furthermore, electroacupuncture significantly reduced levels of Nav 1.8 and COX-2 by western blot analysis, but not vice versa as AM251 treatment. Our data indicate that electroacupuncture mediates antinociceptive effects through peripheral endocannabinoid system signaling pathway and provide evidence that electroacupuncture is beneficial for pain treatment.

## Introduction

Pain is a common problem that affects patients’ quality of life and incurs huge economic costs. The mechanisms of pain are complex, and neuro-immune activation modulates the initiation and maintenance of pain. The on-demand endocannabinoid system has been shown to be involved in various physiological processes. Notably, emerging preclinical and clinical studies have demonstrated that targeting the endocannabinoid system offers opioid-sparing effects in pain treatment [1]. The two main endocannabinoids are N-arachidonoyl ethanolamine (anandamide, [AEA]) [2] and 2-arachidonoylglycerol (2-AG) [3], derivatives of arachidonic acid. Fatty acid amide hydrolase (FAAH) and monoacylglycerol lipase (MAGL) principally metabolize AEA and 2-AG, respectively. Endocannabinoids are endogenous ligands that bind to cannabinoid G-protein-coupled receptors type 1 (CB1) and type 2 (CB2) [4]. Both cannabinoid receptor agonists and inhibitors of FAAH and MAGL have reliable antinociceptive effects.

CB1 is highly expressed throughout the nervous system, modulates the release of neurotransmitters and neuropeptides from presynaptic nerve endings, and inhibits synaptic transmission [5]. Stimulation of CB1 leads to the activation of K+ channels and inhibition of voltage-gated Ca2+ channels and adenylyl cyclase, subsequently downregulating the cAMP/PKA pathway [6]. In contrast, CB2 receptors are mainly expressed in immune cells and peripheral tissues. CB2 activation inhibits adenylyl cyclase activity and stimulates the induction of mitogen-activated protein kinase phosphatase-1 (MKP-1), resulting in the activation of mitogen-activated protein kinase signal transduction [7]. CB2 encompasses immunomodulatory functions and maintenance of synaptic plasticity, and may have potential as a therapeutic target for neuropathic pain and neurodegenerative diseases [8].

Acupuncture has had beneficial effects on pain relief and treatment for more than 2500 years in the East Asian cultural sphere. However, the mechanisms underlying the effects of acupuncture are still not well understood. Analgesia effects of acupuncture are through multidimensional pathways including stimulations of Aδ- and C-fibers [9, 10], meanwhile, releasing of bioactive chemicals such as endorphin [11], adenosine [12], serotonin [13], and endocannabinoids [14]. Electroacupuncture (EA) increases the production of endocannabinoids and its beneficial effects are eliminated by AM251 (a selective CB1 antagonist) in the rat brain [14]. EA is applied to the Huantiao (GB30) and Yanglingquan (GB34) acupoints to reduce inflammatory pain by increasing CB2 levels [15]. It has been reported that EA, applied to Neixiyan (Ex-LE4) and Dubi (ST35), inhibits chronic pain in a mouse model of knee osteoarthritis. Its signaling pathway involves elevated levels of endocannabinoids and CB1 in the ventrolateral periaqueductal gray (vlPAG) [16]. A recent study demonstrated that low-frequency EA stimulation through median nerve induced an anti-nociceptive effect and led to higher orexin A and lower GABA levels in the vlPAG. It suggests that peripheral neurostimulation activates hypothalamic orexin neurons to induce a CB1-dependent cascade [17].

Peripheral neurostimulation of acupuncture through endocannabinoid system, may be one of the primary mediators, has been shown to relieve pain by regulating pain perception levels and anti-inflammatory effects experimentally. Acupuncture regulates the endocannabinoid system and is a novel research topic. However, the effects of EA on CB1 expression in the peripheral nervous system (PNS) have not been elucidated. Peripheral neurostimulation could be a viable target for the treatment of pain. In the present study, we aimed to determine whether EA reduces inflammatory pain by activating CB1 in the dorsal root ganglia (DRG). Dual immunofluorescence staining demonstrated CB1 was co-localized with Nav 1.8, a voltage-gated sodium ion channel that generates and conducts nociceptive action potentials (APs) [18]. Furthermore, blocking CB1 eliminated the analgesic effects and anti-inflammatory pathways. Our data indicate that EA attenuates nociceptive processing through endogenous cannabinoid signaling.

## Materials and methods

### Animals

C57/B6 female mice aged 8–12 weeks were purchased from the BioLASCO Animal Center (BioLASCO, Yilan, Taiwan). Animals were housed under a 12–12-h light–dark cycle and 22°C ambient temperature, food and water were provided ad libitum. All procedures were performed in accordance with the National Institute of Health Guide for the Care and Use of Laboratory Animals. The study protocol was approved by the Ethics Committee of Hualien Tzu Chi Hospital, Hualien, Taiwan (permit No. 110–38 and 110–55).

### Induction of inflammatory pain model and grouping

Mice were anesthetized with 1% isoflurane and were injected with 20 μl of saline or CFA (diluted in the same volume of saline; Sigma, St. Louis, USA) in the right plantar surface of the hind paw to induce intraplantar inflammation. EA was applied using stainless-steel acupuncture needles (1 inch, 32 G, Yu Kuang, Taipei, Taiwan) that were inserted into the muscle layer to a depth of 2–3 mm at the GB30 and GB34 acupoints ipsilaterally under 1% isoflurane anesthetization. Electrical stimuli were delivered using an EA portable stimulator (Ontai, New Taipei City, Taiwan) at an intensity of 1 mA for 15 min at a frequency of 2 Hz. AM251 (Sigma, St. Louis, USA) was dissolved in the dilution solution (dimethyl sulfoxide: Tween-80: saline = 1:1:8) and was injected at GB30 and GB34 acupoints (10 μl/each acupoint, 5 mg/kg body weight total dose) ipsilaterally before EA treatment. This solution was also used as a vehicle control. A total of seventy-two mice were randomly subdivided into eight groups of: (1) Control group: normal saline injection, (2) CFA group: CFA injection to induce inflammatory pain, (3) EA group: CFA injection and EA was applied at GB30 and GB34 acupoints, (4) Sham group: CFA injection and needles inserted at two acupoints without electrical stimulation, (5) Control + vehicle group: normal saline injection and vehicle control injection at two acupoints, (6) CFA + vehicle group: CFA injection and vehicle control injection at two acupoints, (7) EA + vehicle group: CFA injection and vehicle control injection, then EA was applied at two acupoints, and (8) AM251 group: CFA injection and AM251 injection at two acupoints, then EA was applied.

### Nociceptive behavior test

Mechanical and thermal sensitivities were measured at baseline and 24–72 h after the CFA injection. All examinations were performed only when the mice were calm and were not sleeping or grooming. Mechanical sensitivity was measured by examining the force of response to stimulation using three applications of an automated mechanical stimulation apparatus (Ugo Basile SRL, Gemonio, Italy). Thermal sensitivity was measured by examining the latency of responses to stimulation with three applications of thermal stimulation from a Hargreaves apparatus (Ugo Basile SRL, Gemonio, Italy). Briefly, mice were placed on a metal mesh or plexiglass platform for thermal sensitivity, with a plexiglass cage, and habituated for 30 min. The plantar region of the hind paw was stimulated by a tip or focused light source for thermal sensitivity, and force Gram counts or withdrawal latencies for thermal sensitivity were automatically recorded when the stimulation caused the mouse to withdraw the paw.

### Immunofluorescence staining

After measuring of behavior tests at 72 h, mice were sacrificed using CO2 exposure. The right side lumbar DRG L3-L5 were immediately dissected and were post-fixed with 4% paraformaldehyde at 40C for 3 days. The tissues were then placed in 30% sucrose (w/v) for cryoprotection. The DRG were embedded in frozen section media (Sakura Finetek, CA, USA) and were cut into 10-um sections in cryostat (Leica Biosystems, Nussloch, Germany). The sections were fixed with 4% paraformaldehyde for 30 min and were rinsed with 0.05% Tween 20/phosphate buffered saline (PBS-T) three times, then were blocked with 5% BSA, 0.1% Triton X-100, and 0.01% sodium azide for 30 min at room temperature. The sections were incubated with primary antibody Nav 1.8 (1:200) (Alomone, Jerusalem, Israel), the CB1 (1:100) (Santa Cruz Biotechnology, Texas, USA) at 4°C overnight in a moisture chamber. The secondary antibodies were incubated with goat anti-rabbit IgG DyLight488 (1:1000) (KPL, MD, USA) and goat anti-mouse IgG DyLight680 (1:1000) (KPL, MD, USA) for 2 hours at 4°C. The stained samples were mounted with a fluorescent mounting medium (KPL, MD, USA), sealed under a coverslip, and photographed using a fluorescent microscope (Axio Observer 7, Carl Zeiss Microscopy, NY, USA).

### Western blot analysis

As mentioned above, the right side L3-L5 DRG were immediately dissected and were stocked at -80°C. Total proteins were prepared by homogenization with lysis buffer (NP40 cell lysis buffer; Thermo Fisher Scientific, Vienna, Austria) containing a protease inhibitor (Thermo Fisher Scientific, IL, USA) and PMSF (final concentration of 1 mM; Sigma, St. Louis, USA). Ten micrograms of protein from each sample were analyzed using a BCA protein assay. The extracted proteins were subjected to 8–12% SDS-Tris glycine gel electrophoresis and transferred onto a PVDF membrane. The membrane was blocked with 5% nonfat milk in a PBS-T buffer, incubated with primary antibody Nav 1.8 (1:500) (Alomone, Jerusalem, Israel), CB1 (1:200) (Santa Cruz Biotechnology, Texas, USA), the COX-2 (1:200) (Santa Cruz Biotechnology, Texas, USA), and α-tubulin (1:1000) (Santa Cruz Biotechnology, Texas, USA) in PBS with 1% BSA and 0.01% sodium azide for overnight at 4°C. A peroxidase-conjugated secondary antibody (1:5000) (KPL, MD, USA) was used as the secondary antibody. The protein bands on the membranes were visualized using a Western Chemiluminescent Kit (HyECL, Taipei, Taiwan) with a UVP ChemStudio Touch System (Analytik Jena, CA, USA). The image intensities of specific bands were quantified using NIH ImageJ software (Bethesda, MD, USA).

### Statistical analysis

Statistical analyses were performed using OriginPro 8 software (OriginLab, MA, USA). All statistical data are presented as the mean ± standard error (SEM). Statistical significance was analyzed using one-way ANOVA, followed by the Bonferroni post hoc test. Statistical significance was set at p < .05.

## Results

### The effect of EA treatment on mechanical and thermal pain behaviour

**Fig 1:**
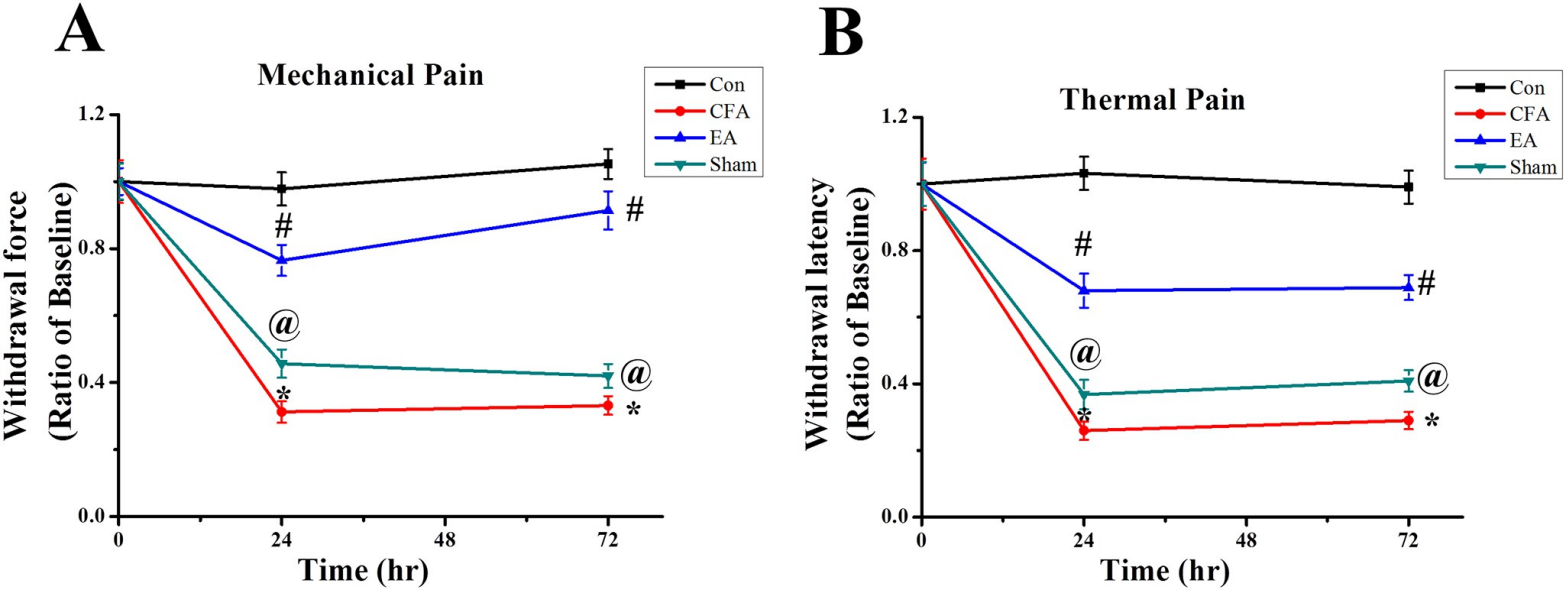
Comparative graphs of mechanical and thermal pain behaviors at baseline 24 h, and 72 h. CFA was injected at the hind paw and nociceptive withdrawal force or latency was measured ipsilaterally. EA was applied at baseline, 24 h, and 72 h under 1% isoflurane anesthetization. (A) Mechanical and (B) thermal pain sensitivities are shown as ratios, compared with baseline for each group (n = 9). * p < .05 for means when compared with control group. p < .05 for means when compared with CFA group. @ p < .05 for means when compared with EA group.

The efficacy of EA in ameliorating inflammatory pain was assessed by comparing the mechanical and thermal pain responses at baseline and at 24 and 72 h. As shown in Fig 1A and 1B, no significant differences were observed among the four groups under basal conditions. Complete Freund’s adjuvant (CFA) injections reduced both mechanical and thermal sensitivities in the CFA, EA, and Sham groups (24 h mechanical sensitivity: 0.32 ± 0.03, 0.77 ± 0.05, and 0.46 ± 0.04, respectively; thermal sensitivity: 0.25 ± 0.02, 0.67 ± 0.05, and 0.38 ± 0.05, respectively) (72 h mechanical sensitivity: 0.33 ± 0.03, 0.91 ± 0.06, and 0.42 ± 0.04, respectively; thermal sensitivity: 0.29 ± 0.03, 0.69 ± 0.04, and 0.41 ± 0.04, respectively). EA applied at GB30 and GB34 significantly reversed the pain phenomena when compared to the CFA and Sham groups at 24 and 72 h. Behavioral nociceptive responses of Sham EA (without electrical stimulation) did not significantly improve compared with those of the EA group.

### EA promoted expression of CB1 in the DRG

**Fig 2:**
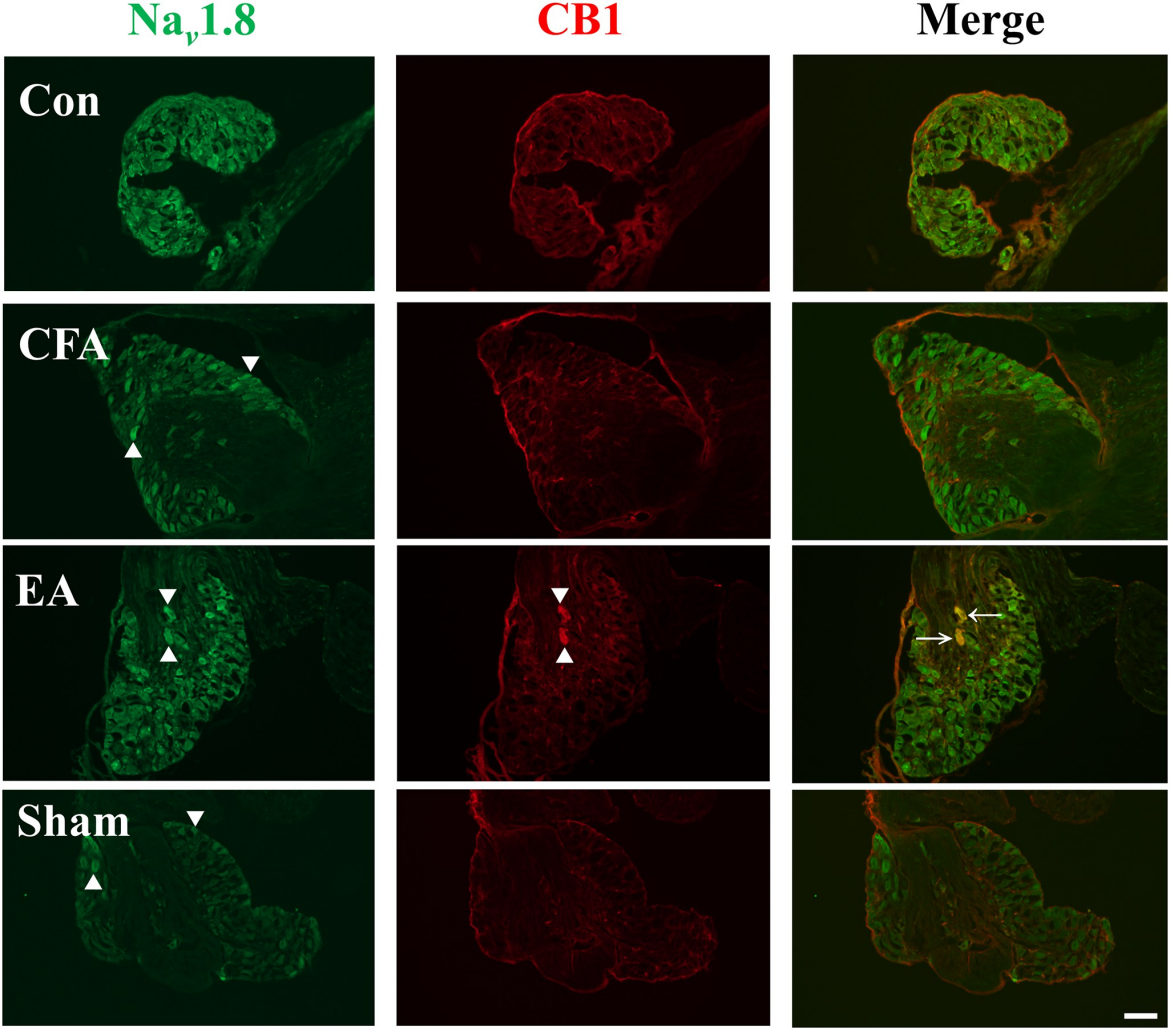
Nav 1.8 and CB1 expression was detected using dual immunofluorescence staining in the DRG. Nav 1.8 immunoreactive cells (green) and CB1 immunoreactive cells (red) were merged (yellow, marked by arrowhead). CFA injection increased expression of Nav 1.8 (marked by triangle) and EA raised expression of CB1 (marked by triangle) (scale bar = 100 *μ*m, n = 3).

We aimed to determine whether EA relieves pain through the analgesic activity of CB1 in the PNS. Mouse DRG were immediately dissected and further visualized by dual immunofluorescence staining 72 h after CFA administration. CFA injection resulted in upregulation of Nav 1.8, but did not alter expression of CB1, compared with the control group (Fig 2, CFA group). It is a high-profile finding that EA elevated expression of CB1 and cell count of co-localization with Nav 1.8, compared with the control, CFA, and sham groups (Fig 2, EA group).

### EA raised levels of CB1 and reduced levels of Nav 1.8

**Fig 3:**
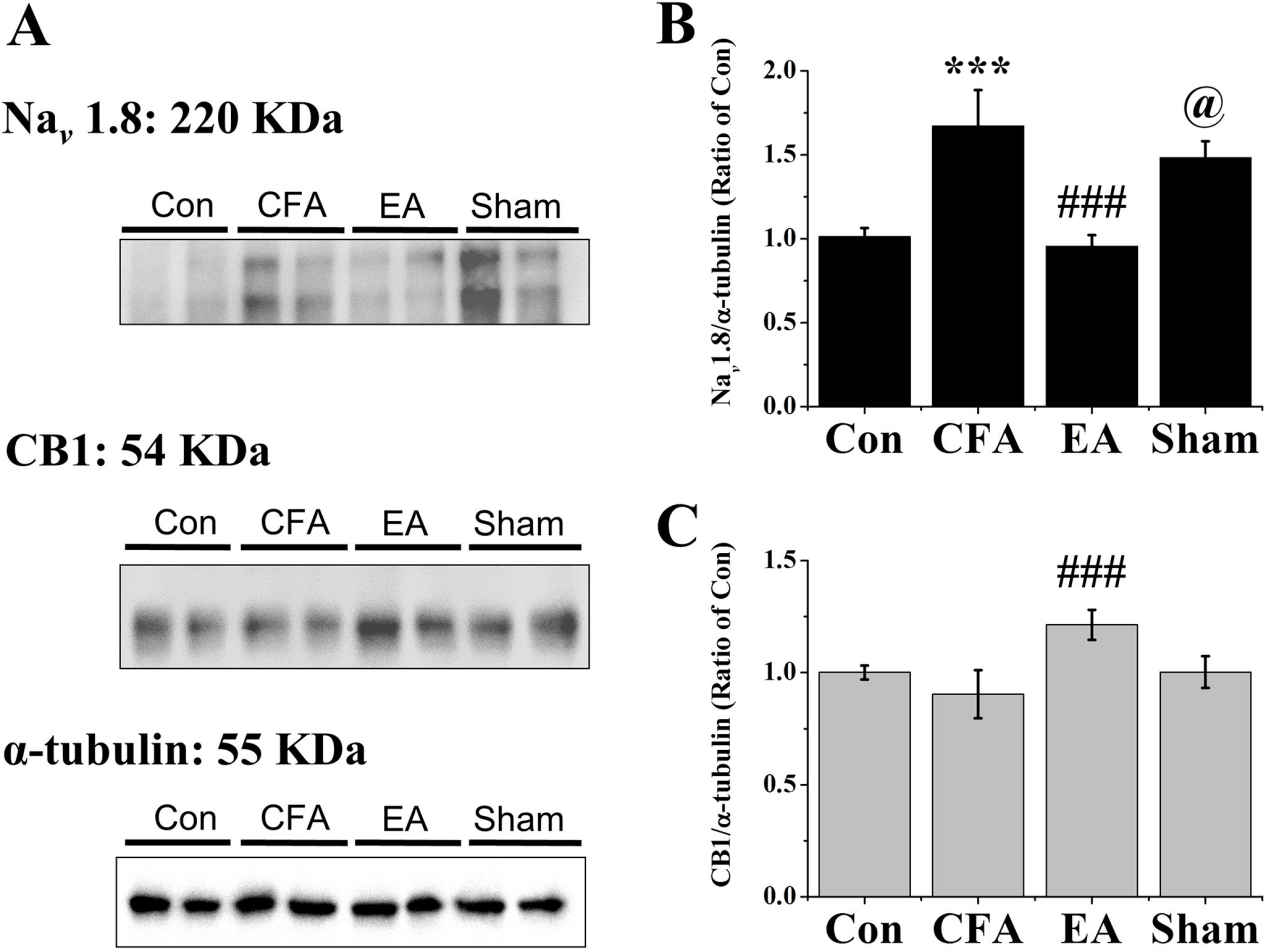
Expression levels of CB1 and Nav 1.8 in the DRGs. (A) Mice DRG homogeneous lysates were immunoreactive with specific antibodies to Nav 1.8, CB1, and α-tubulin (B) Levels of Nav 1.8, (C) Levels of CB1 were normalized with internal control α-tubulin and were compared to that of the control group (n = 6). *** p < .001 for means when compared with control group. p < .001 for means when compared with CFA group. @ p < .05 for means when compared with EA group.

We further assessed protein levels of CB1 and Nav 1.8 using western blot analysis. CFA injection caused overexpression of Nav 1.8, and EA significantly reversed it (CFA:1.67 ± 0.21, EA:0.96 ± 0.07, Sham:1.48 ± 0.10; CFA vs. EA, p < .001) (Fig 3A and 3B). In the Sham EA group, overexpression of Nav 1.8 was similar to the CFA group. In addition, EA significantly raised levels of CB1, Sham EA had no the same effect (CFA:0.90 ± 0.11, EA:1.21 ± 0.07, Sham:1.00 ± 0.07; CFA vs. EA, p < .05) (Fig 3A and 3C).

### A CB1 inhibitor blocked pain relieving effects of EA on mechanical and thermal pain behaviors

**Fig 4:**
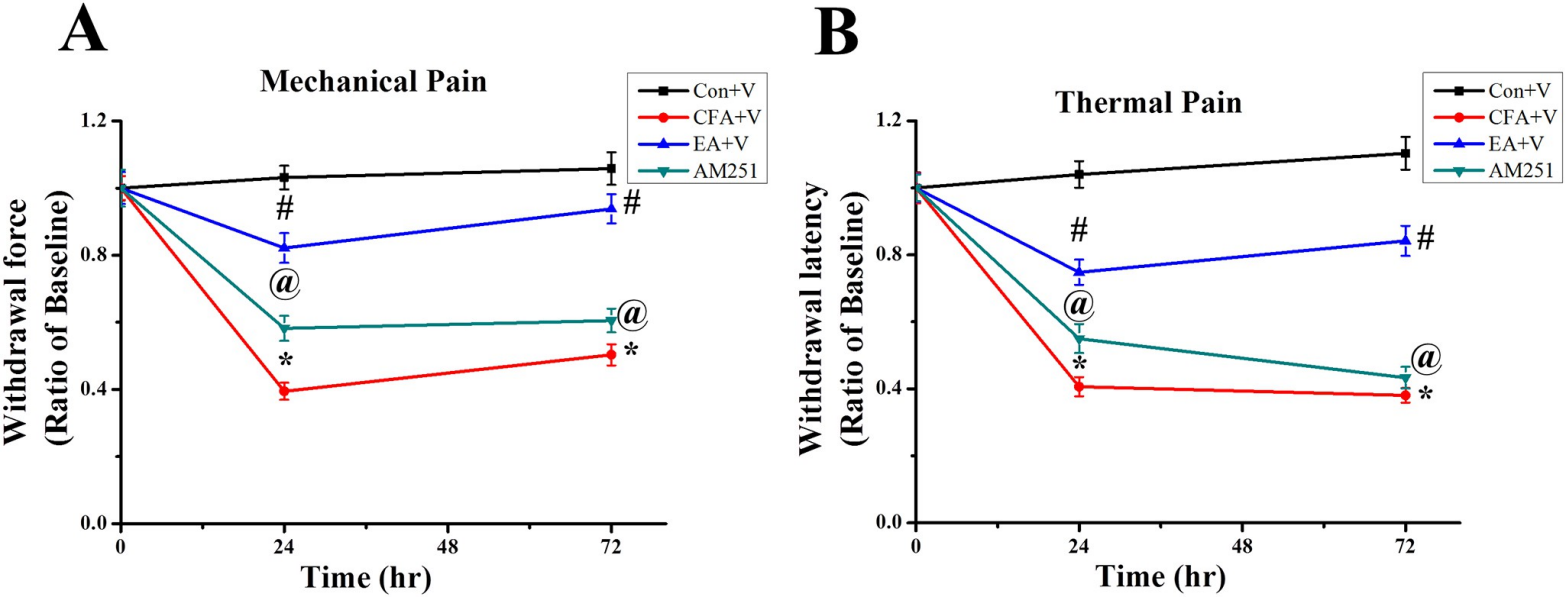
AM251 blocked pain relieving effects of EA on mechanical and thermal pain behaviors. (A) Mechanical and (B) thermal pain sensitivities are shown as ratios compared with the baseline of each group (n = 9). * p < .05 for means when compared with control+V group. p < .05 for means when compared with CFA+V group. @ p < .05 for means when compared with EA+V group.

To investigate the role of CB1 involvement in EA-mediated pain relief, AM251 was injected at GB30 and GB34 (10 μl at each acupoint, 5 mg/kg total quantity) before EA was applied. An equal volume of diluted vehicle was injected at GB30 and GB34 in the control+V, CFA+V, and EA+V groups. Our data revealed no significant differences between the four groups at baseline (Fig 4A and 4B). CFA injection reduced the mechanical and thermal pain threshold in the CFA+V, EA+V, and AM251 groups (24 h mechanical sensitivity: 0.41 ± 0.02, 0.80 ± 0.04, and 0.61 ± 0.04, respectively; thermal sensitivity: 0.41 ± 0.03, 0.75 ± 0.04, and 0.55 ± 0.04, respectively) (72 h mechanical sensitivity: 0.52 ± 0.03, 0.92 ± 0.04, and 0.63 ± 0.04, respectively; thermal sensitivity: 0.39 ± 0.02, 0.84 ± 0.04, and 0.44 ± 0.03). EA significantly ameliorated pain compared to the CFA+V group at 24 and 72 h. Furthermore, AM251 treatment significantly blocked the EA-mediated pain relieving effects compared with the EA+V group (Fig 4A and 4B).

### AM251 treatment reduced expression of CB1 mediated by EA in the DRGs

**Fig 5:**
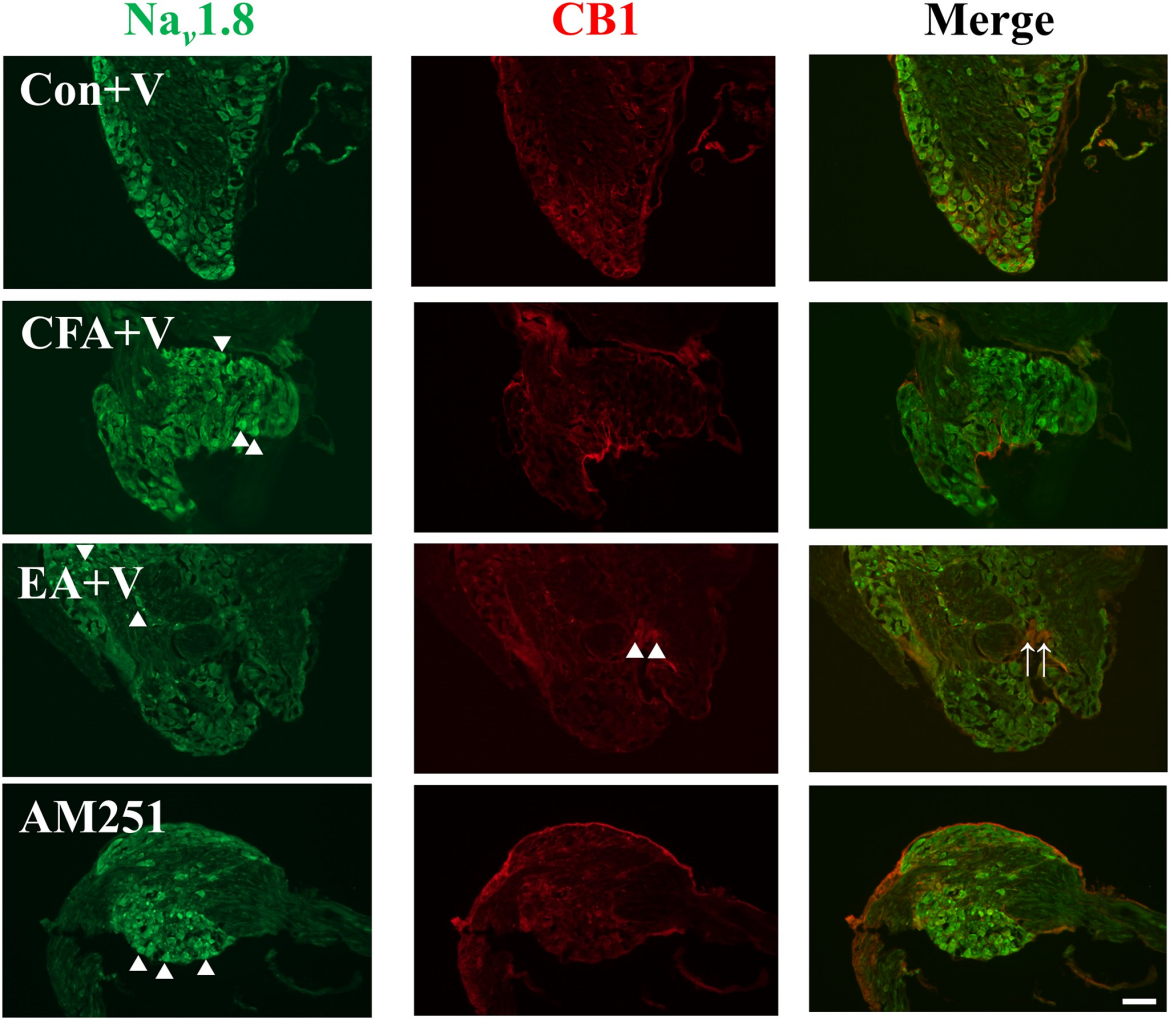
AM251 treatment blocked cellular modulation effects of Nav 1.8 and CB1 mediated by EA in the DRGs. Nav 1.8 immunoreactive cells (green) and CB1 immunoreactive cells (red) were merged (yellow, marked by arrowhead). CFA injection raised expression of Nav 1.8 in the CFA+V and AM251 group (marked by triangle). EA raised expression of CB1 (marked by triangle) and decreased expression of Nav 1.8 in the EA+V group (scale bar = 100 *μ*m, n = 3).

As mentioned above, mouse DRG were immediately dissected 72 h after CFA injection. CFA up-regulated cellular expression of Nav 1.8 in the CFA+V and sham+V group and had no change in CB1 expression compared with the control+V group. Our results showed that EA increased CB1 expression and decreased Nav 1.8 expression compared to the CFA+V group (Fig 5, EA+V group). AM251 treatment did not reduce the number of Nav 1.8 and blocked a raising effect of CB1 immunoreactive cells mediated by EA compared with EA+V group (Fig 5, AM251 group). AM251 blocked the cellular modulation effects of EA in the expression of Nav 1.8 and CB1.

### AM251 treatment blocked EA-mediated down-regulation of Nav 1.8 and an increasing of CB1

**Fig 6:**
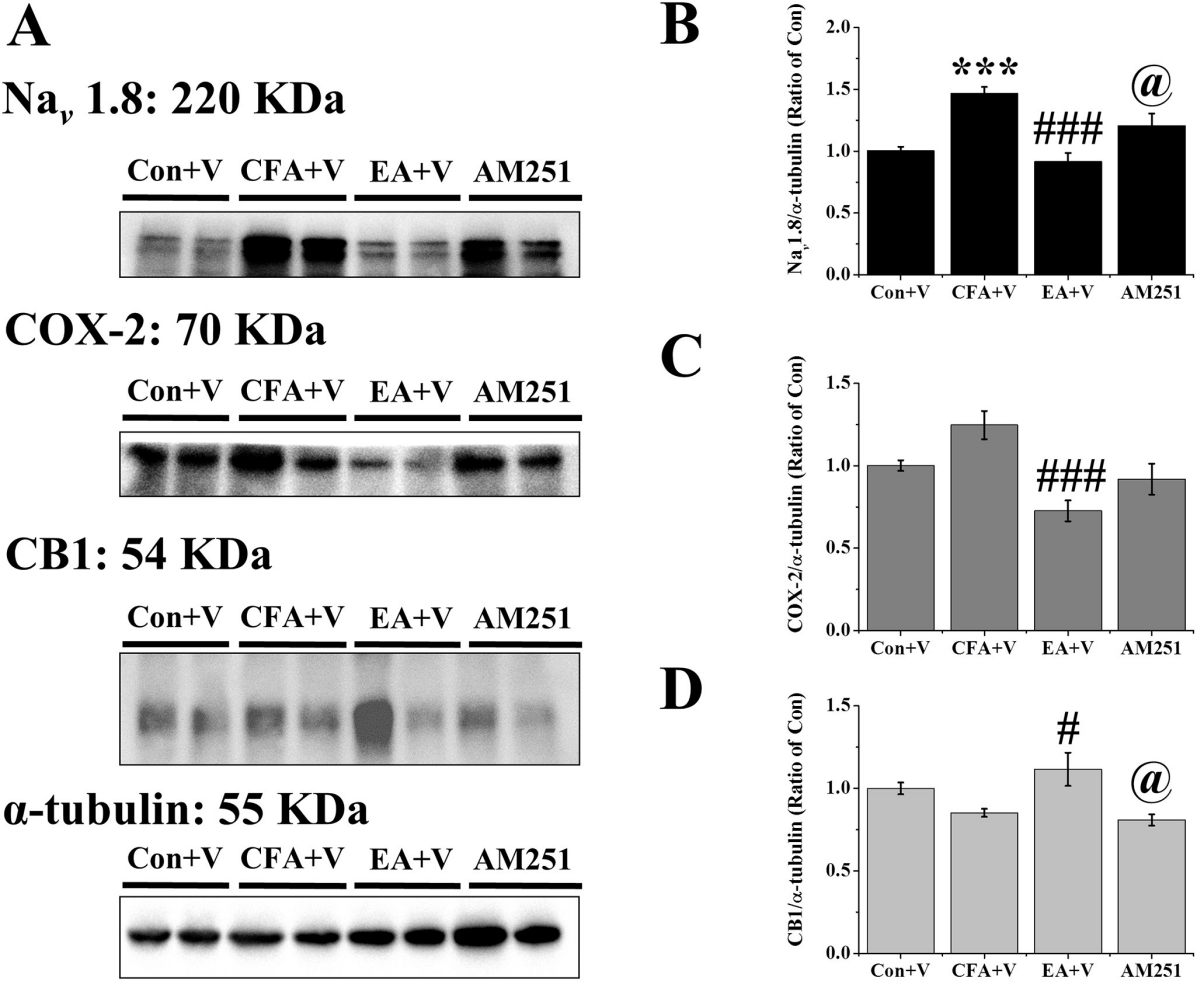
Expression levels of Nav 1.8, COX-2, and CB1 in the DRG. (A) Mice DRGs homogeneous lysates were immunoreactive with specific antibodies to Nav 1.8, COX-2, CB1, and α-tubulin. (B) Levels of Nav 1.8, (C) Levels of COX-2, (D) Levels of CB1 were normalized with internal control α-tubulin and were compared to that of the control group (n = 6). *** p < .001 means when compared with control+V group. p < .05, p < .001 for means when compared with CFA+V group. @ p < .05 for means when compared with EA+V group.

We further assessed protein levels of Nav 1.8, COX-2, and CB1 using western blot analysis. EA significantly reduced protein levels of Nav 1.8 (CFA+V: 1.47 ±0.05, EA+V: 0.92 ± 0.07, AM251: 1.21 ± 0.10; CFA+V vs. EA+V, p < .001) (Fig 6A and 6B) and COX-2 (CFA+V: 1.25 ± 0.09, EA+V: 0.73 ± 0.06, AM251: 0.92 ± 0.09; CFA+V vs. EA+V, p < .001) (Fig 6A and 6C). Furthermore, AM251 injection significantly blocked an increase of CB1 (CFA+V: 0.85 ± 0.02, EA+V: 1.12 ± 0.10, AM251: 0.81 ± 0.03 EA+V vs. AM251, p < .001) (Fig 6A and 6D) and down-regulation of Nav 1.8 (EA+V vs. AM251, p < .05) (Fig 6A and 6B). It has partial blocked effects of COX-2 (EA+V vs. AM251, p = .45) (Fig 6A and 6C).

## Discussion

In this study, we showed that EA significantly increased CB1 expression in the DRG. AM251 eliminated EA-mediated anti-nociceptive and immunomodulatory effects. These findings demonstrate that EA attenuates inflammatory pain via the peripheral CB1 endocannabinoid signaling pathway. To the best of our knowledge, no previous studies have examined the relationship between EA and CB1 in the PNS. Our data confirm that peripheral CB1 plays a pivotal role in EA-mediated anti-nociception. Activation of CB1 and CB2 has been shown to ameliorate the induction and maintenance of inflammatory pain [19, 20]. CB1 knockout mice displayed increased hypoalgesia during mechanical and heat noxious stimuli. This suggests that CB1 exerts analgesic effects during pain processing [21]. Endocannabinoids and CB1 are believed to attenuate excitotoxicity of neuropathic insults. Cannabinergic modulation protects against neuronal damage and facilitates tonic signaling by binding to CB1 [22, 23]. CB1 is abundantly expressed in primary afferent peripheral nerves, including in the cell bodies of the trigeminal ganglion and DRG, and mediates an inhibitory tone for nociceptive activity. Several studies have shown that the peripheral terminals of nociceptors are important sites for cannabinergic circuit modulation [24–26]. Interestingly, acupuncture seems to activate both of CB1 and CB2. Administration of AM630 (a CB2 antagonist with 70- to 165-fold selectivity in vitro) before EA treatment blocked anti-inflammatory effects [27]. Our present data showed that AM251 could not completely block EA-mediated down-regulation of COX-2. The possible mechanism may due to EA drive the vagal–adrenal anti-inflammatory axis [28]. CB1 is primarily responsible for pain perception, thus CB2 moderates the anti-inflammatory effects of acupuncture.

Recently, cannabinoids have been shown to be a potential therapeutic agent for reducing opioid use for inflammatory and neuropathic pain. However, in humans, systemic administration of cannabinoids causes psychotropic, memory impairment, and motor control side effects, which have largely limited their clinical use [1, 29]. A major challenge in the clinical use of cannabinoids is reducing or eliminating their adverse effects without attenuating their analgesic effects. The peripheral endocannabinoid system is an important component of pain control mechanisms. The activation of peripheral CB1 can induce cannabinoid-induced analgesia without causing any central side effects [24, 30]. In addition, our results provide a novel idea that EA stimulates peripheral CB1 signaling pathway to relieve inflammatory pain and hyperalgesia.

Dysregulation of the endogenous cannabinoid system has been demonstrated in several chronic diseases that affect females, such as fibromyalgia and multiple sclerosis [31, 32]. Cannabinoids have better mechanical and heat pain-beneficial responses in female rats than those in males [33, 34]. The sexually dimorphic effects of the cannabinoid system may be generalized based on the CB receptor-binding properties, endocannabinoid levels, and pharmacokinetics of cannabinoids [35]. Sex steroids are important modulators of CB receptor expression. Male rats exhibited higher levels of CB1 mRNA in the pituitary gland than female rats, and orchiectomy of male rats reduced CB1 mRNA levels. Moreover, estrogen decreased CB1 mRNA expression in ovariectomized rats [36]. CB1 levels were decreased in castrated male rats and were restored following testosterone replacement [37]. Inflammatory pain–induced upregulation of CB receptors also shows sex-related differences. Males display a greater enhancement of CB1 under conditions of inflammatory cytokine activation [38]. These studies suggest that sex hormones and inflammation can influence endogenous cannabinoid tone. Sex differences in the peripheral administration of cannabinoids should be considered critically.

Functional interactions between CB1 and transient receptor potential vanilloid 1 (TRPV1) receptor mediate analgesia by the administration of acetaminophen, an analgesic and antipyretic drug, suggesting indirect activation of CB1 [39]. Importantly, peripheral norepinephrine release from sympathetic terminals is controlled by CB1. Crosstalk between CB1 and TRPV1 modulates anti-inflammatory pain [40]. Analgesic effects of systemically administered cannabinoids are strongly reduced by using a conditional knockout CB1 technique under the control of the Nav 1.8 promoter [24]. A recent study confirmed that cannabidiol binds selectively to Nav 1.8 channels contributing to its analgesic effects [41]. Furthermore, knocking out CB1 in GABAergic and glutamatergic neurons in the PAG abolished EA-induced analgesic effects [42]. EA mediates analgesic mechanisms including suppression of Nav 1.8 and TRPV1 through neuronal and non-neuronal pathways [43]. Our data show that co-expression of CB1 and Nav 1.8 in DRG suggests an antinociceptive cross regulation between the two receptors. These studies implicate EA targeting peripheral CB1 to Nav 1.8 provides effective pain relief.

There were several limitations to our study. First, in this animal study, AM251 was performed in GB30 and GB34 acupoints by intramuscular injection. AM251 is able to cross the blood–brain barrier. Systemic and central effects of AM251 should be considered. Second, how EA leads to increasing of CB1 in DRG remains to be clarified. Third, whether this analgesic mechanism in humans is uncertain and needs more clinical studies.

## Conclusion

In conclusion, we demonstrated that EA triggers peripheral CB1 endocannabinoid signaling to relieve inflammatory pain. EA elevated expression of CB1 co-localization with Nav 1.8, and further reduced hyperalgesia and inflammation. These phenomena were eliminated by AM251 treatment and confirmed an EA-mediated peripheral CB1 antinociceptive signaling mechanism. Our data indicated that EA increased the production of cannabinoid analgesia, inhibited nociceptive processing, and provided evidence that EA could be useful for successful pain treatment.

## Supporting information

S1 File The data set. Raw data of relevant experiments in the manuscript.
(XLSX)

S2 File Western blot results. Original pictures of the western blot analysis in the manuscript.
(ZIP)
