# Supplementary figures and images for: Electroacupuncture attenuates inflammatory pain via peripheral cannabinoid receptor type 1 signaling pathway in mice
Source: PLoS One. 2023 Dec 7;18(12):e0295432. doi: 10.1371/journal.pone.0295432 (PMC10703209; doi:10.1371/journal.pone.0295432)

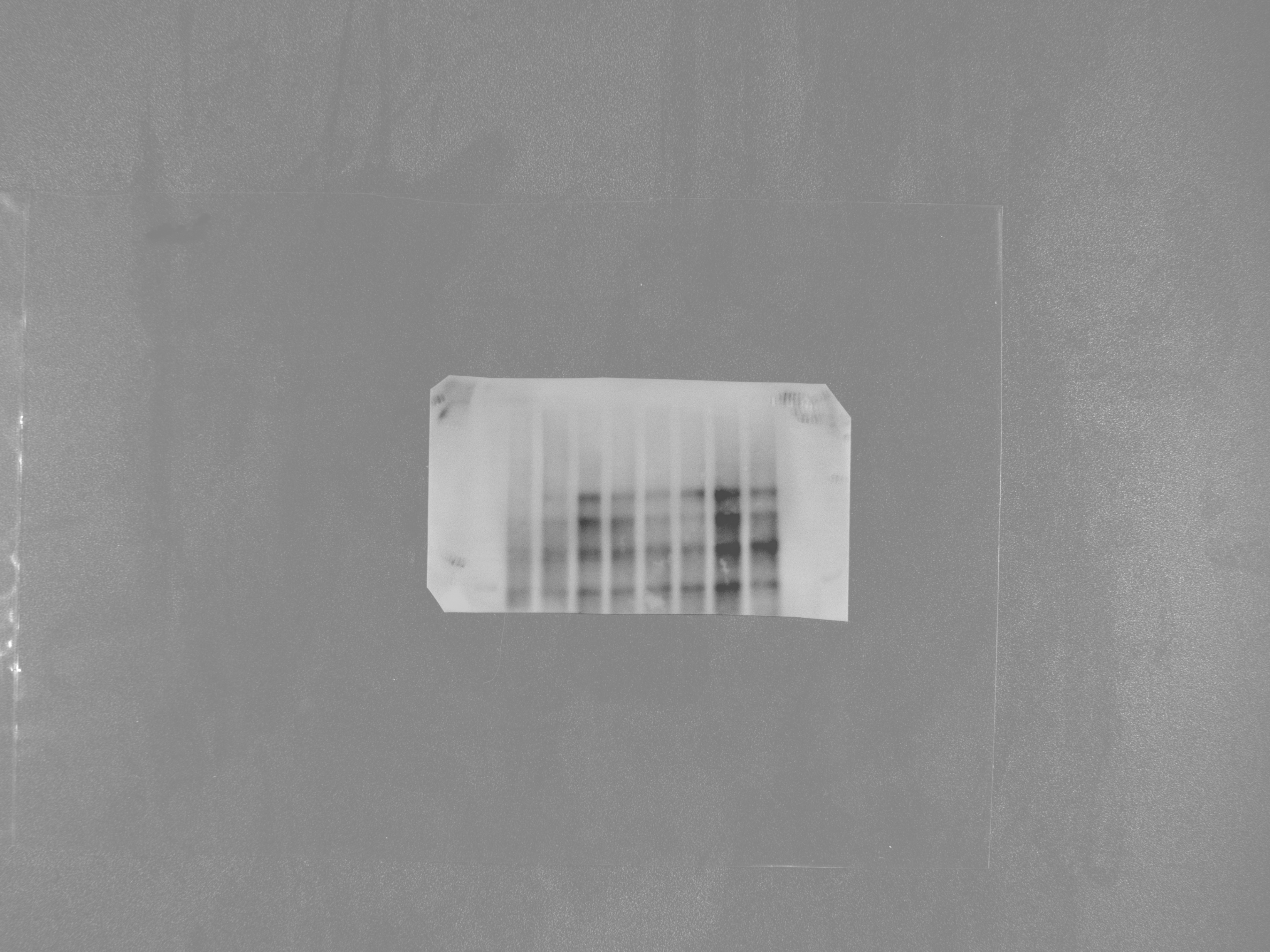

Supplement: S2 File — Original pictures of the western blot analysis in the manuscript. (ZIP) [file pone.0295432.s002.zip › Western blot results/Fig 3 B-Nav18-1.tif]

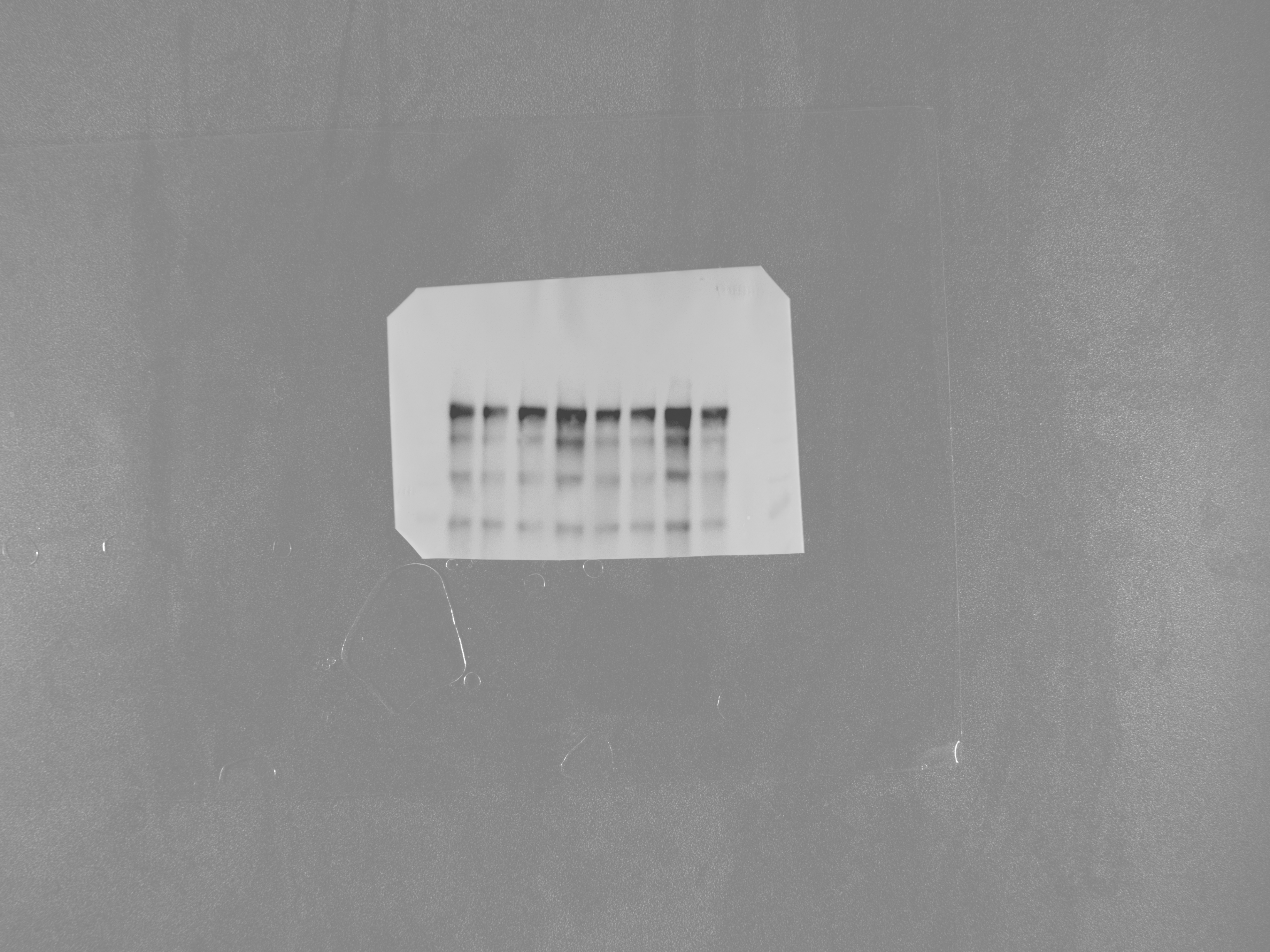

Supplement: S2 File — Original pictures of the western blot analysis in the manuscript. (ZIP) [file pone.0295432.s002.zip › Western blot results/Fig 3 B-Nav18-2.tif]

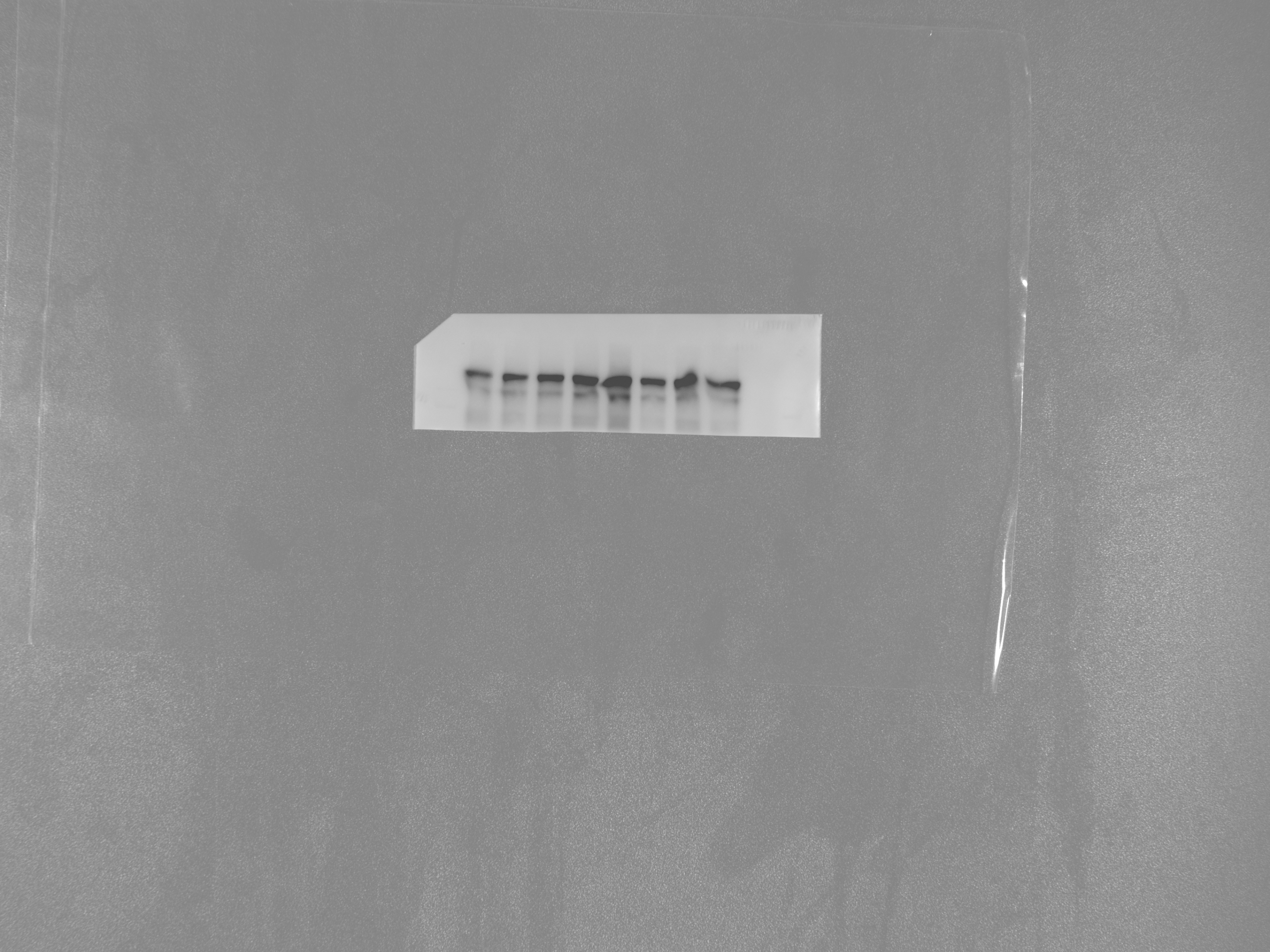

Supplement: S2 File — Original pictures of the western blot analysis in the manuscript. (ZIP) [file pone.0295432.s002.zip › Western blot results/Fig 3 B-Nav18-3.tif]

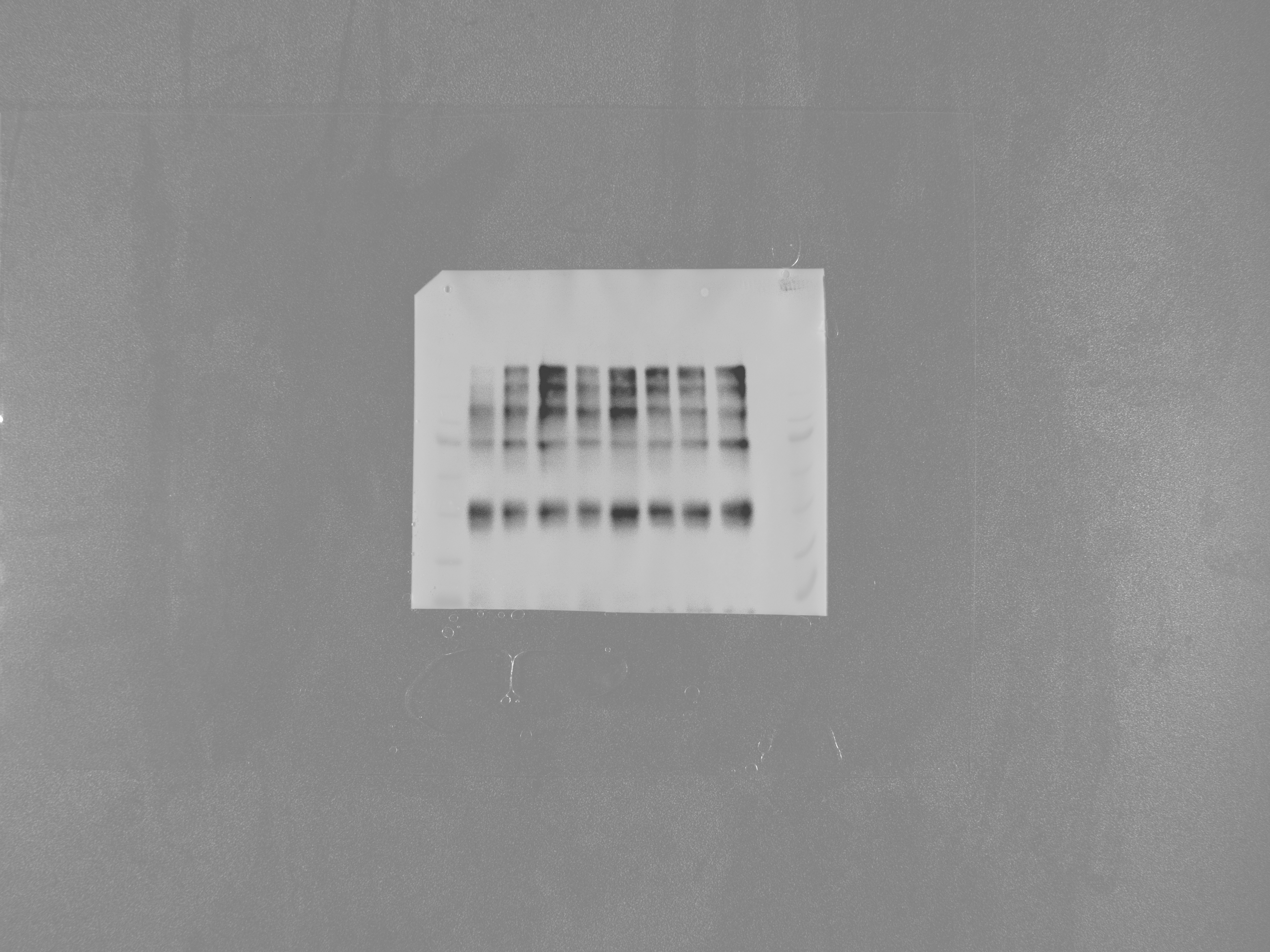

Supplement: S2 File — Original pictures of the western blot analysis in the manuscript. (ZIP) [file pone.0295432.s002.zip › Western blot results/Fig 3 C-cb1-1.tif]

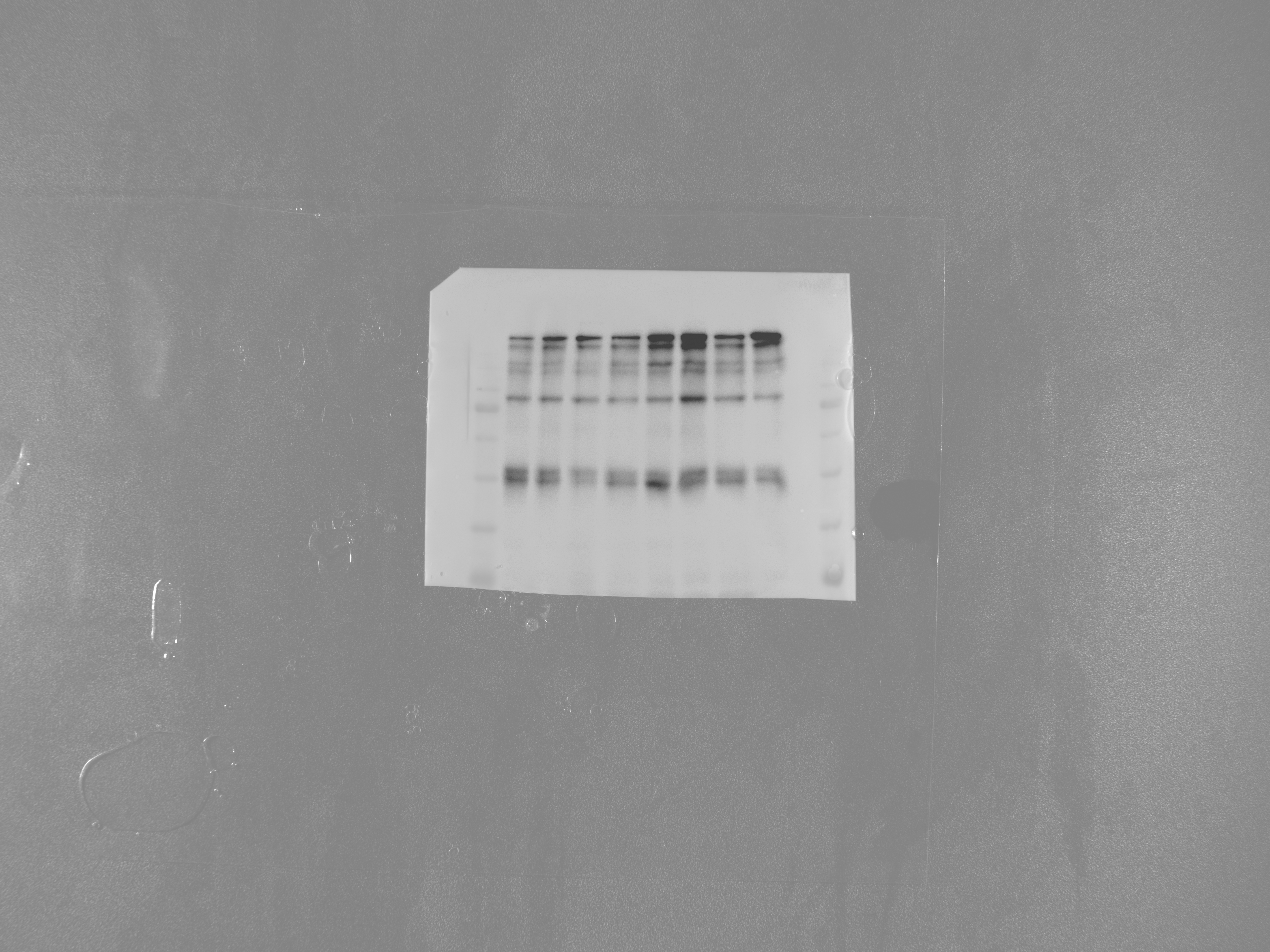

Supplement: S2 File — Original pictures of the western blot analysis in the manuscript. (ZIP) [file pone.0295432.s002.zip › Western blot results/Fig 3 C-cb1-2.tif]

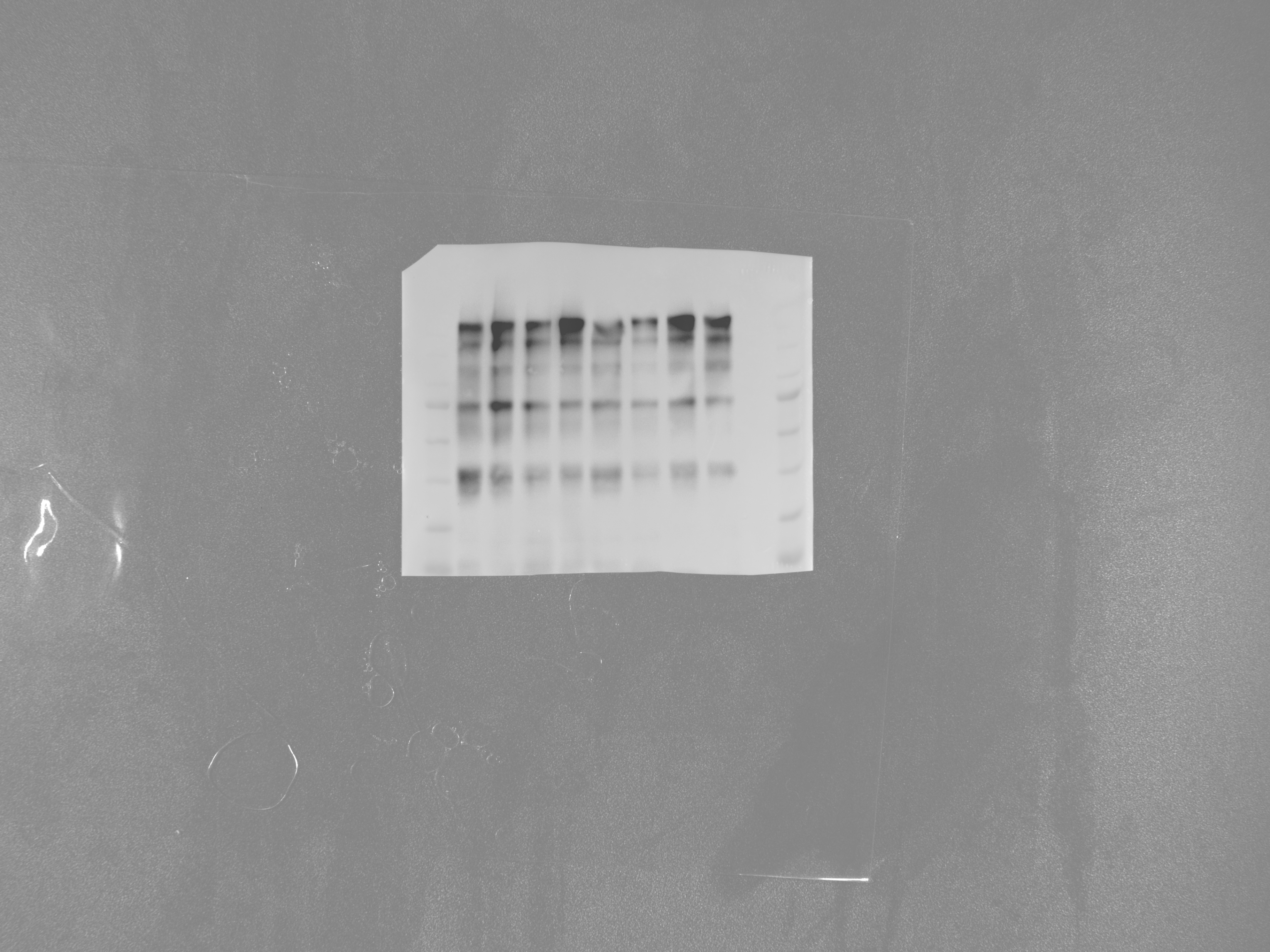

Supplement: S2 File — Original pictures of the western blot analysis in the manuscript. (ZIP) [file pone.0295432.s002.zip › Western blot results/Fig 3 C-cb1-3.tif]

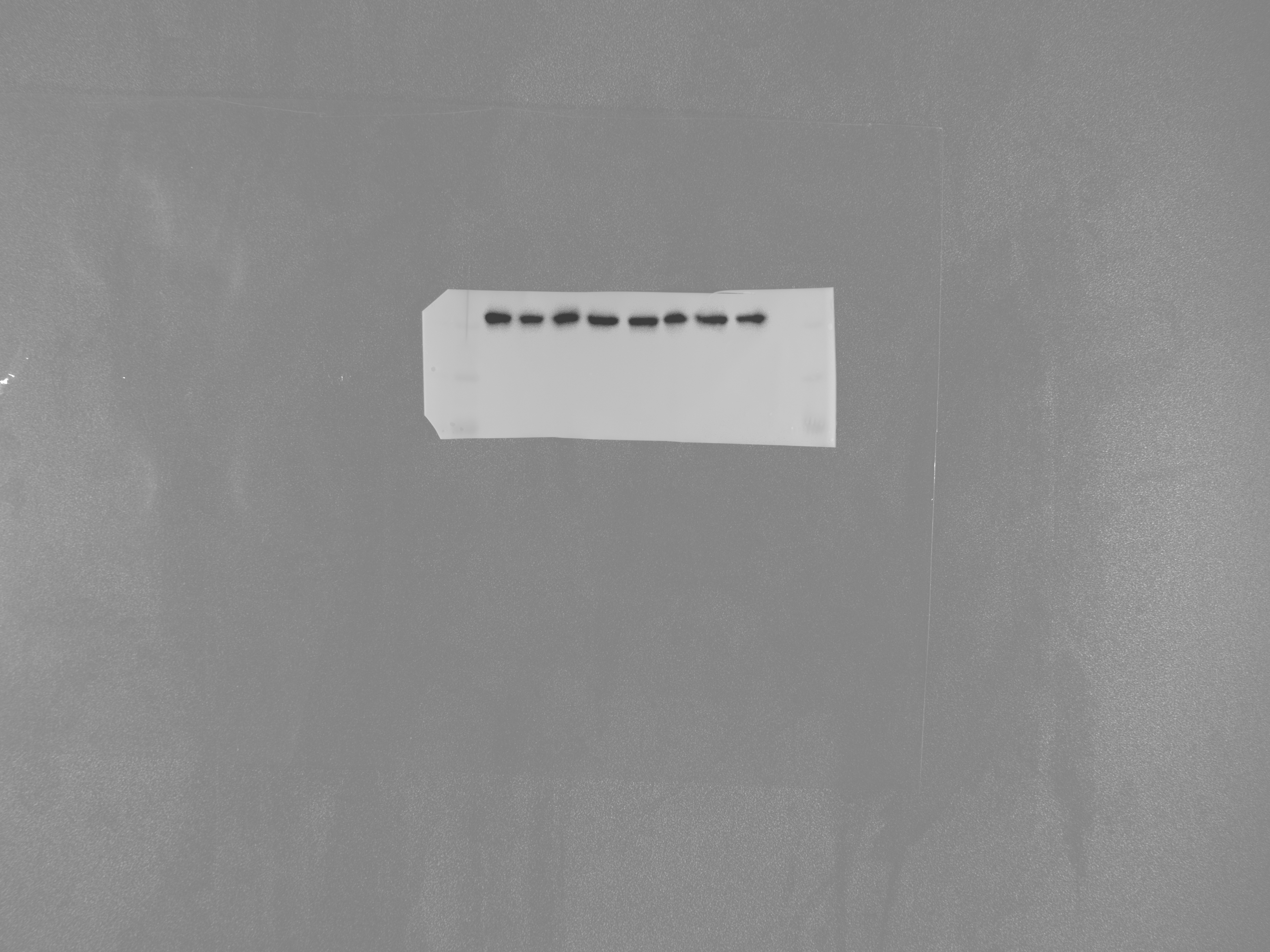

Supplement: S2 File — Original pictures of the western blot analysis in the manuscript. (ZIP) [file pone.0295432.s002.zip › Western blot results/Fig 3-tubulin-1.tif]

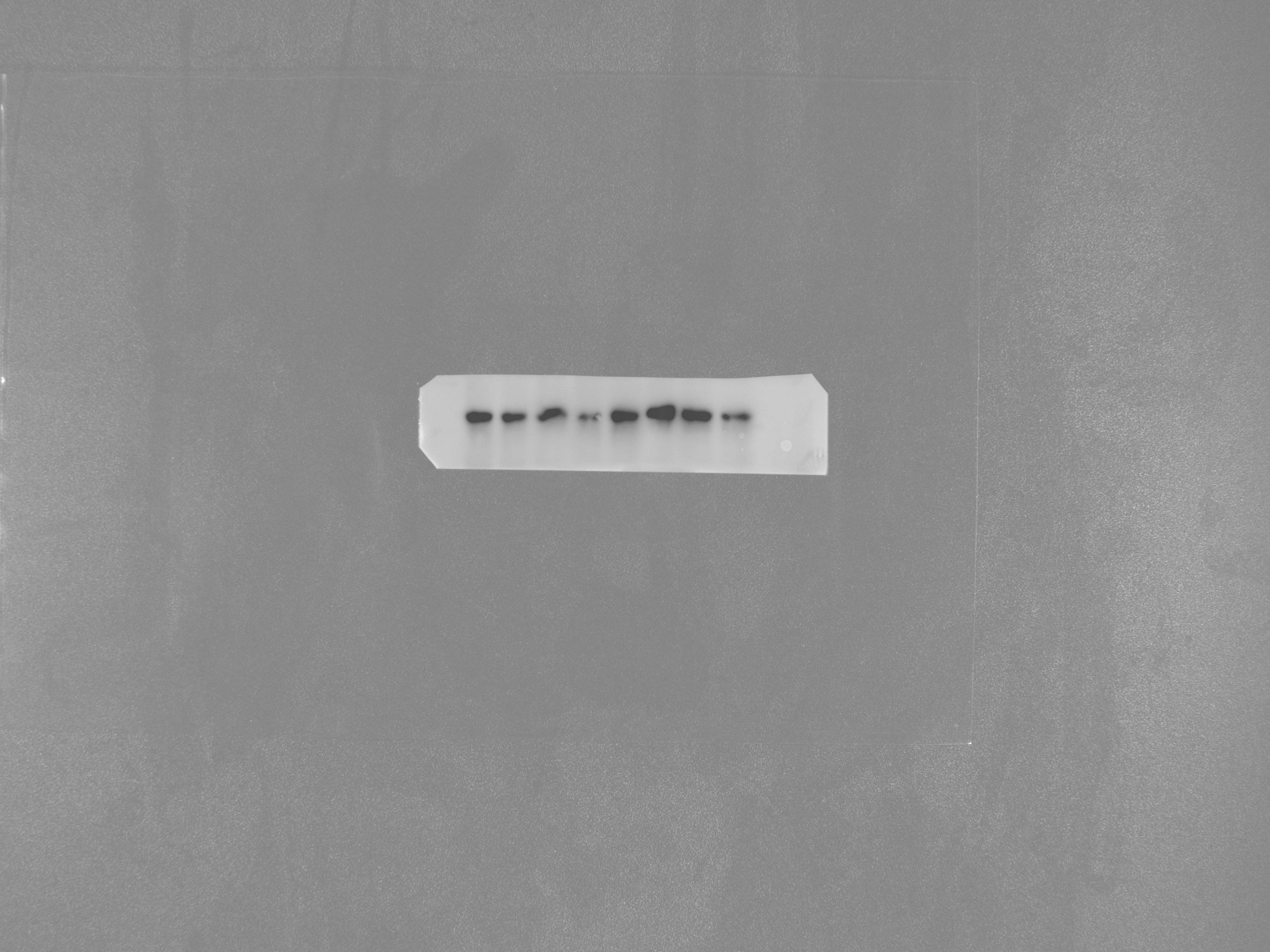

Supplement: S2 File — Original pictures of the western blot analysis in the manuscript. (ZIP) [file pone.0295432.s002.zip › Western blot results/Fig 3-tubulin-2.tif]

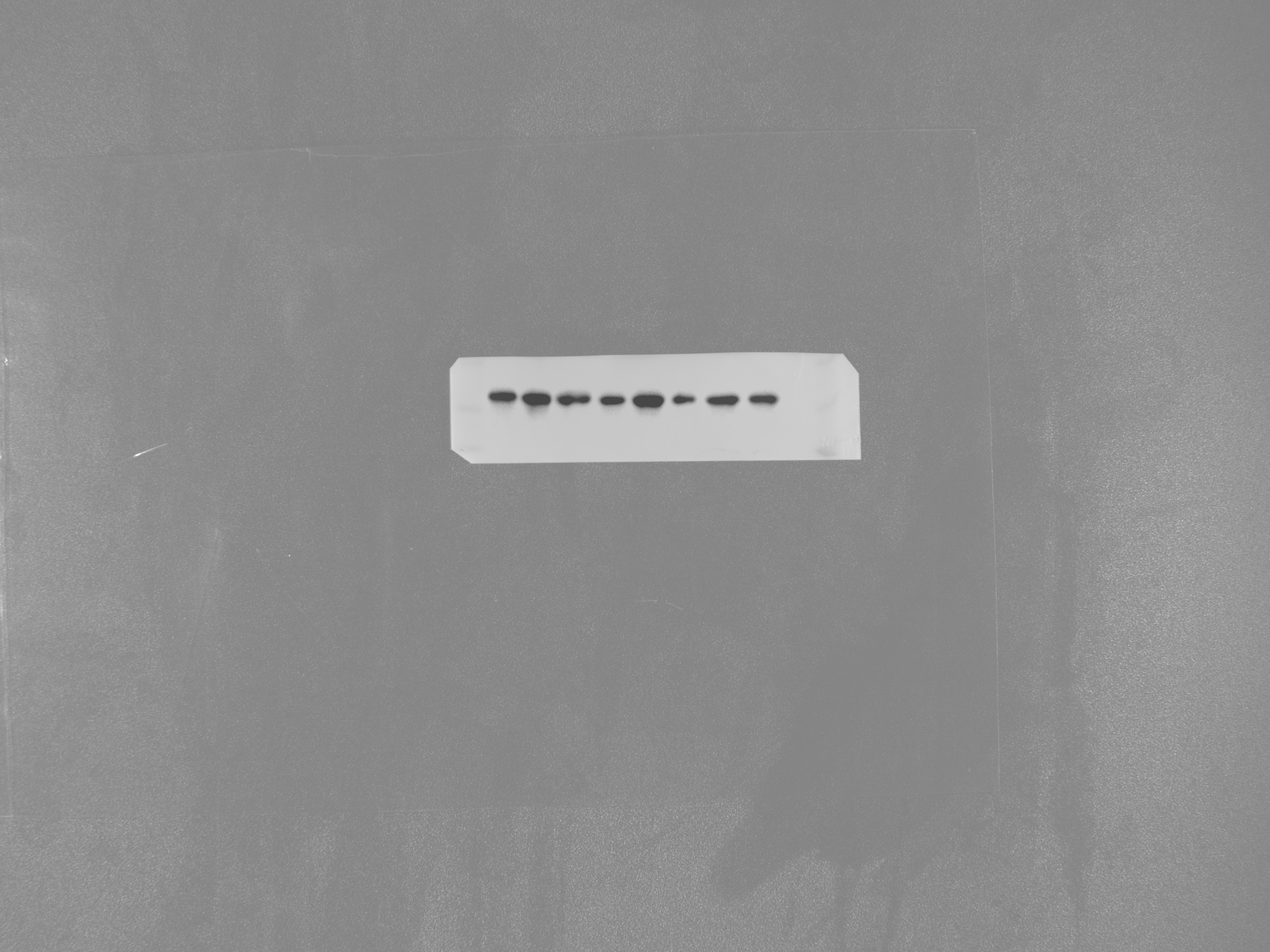

Supplement: S2 File — Original pictures of the western blot analysis in the manuscript. (ZIP) [file pone.0295432.s002.zip › Western blot results/Fig 3-tubulin-3.tif]

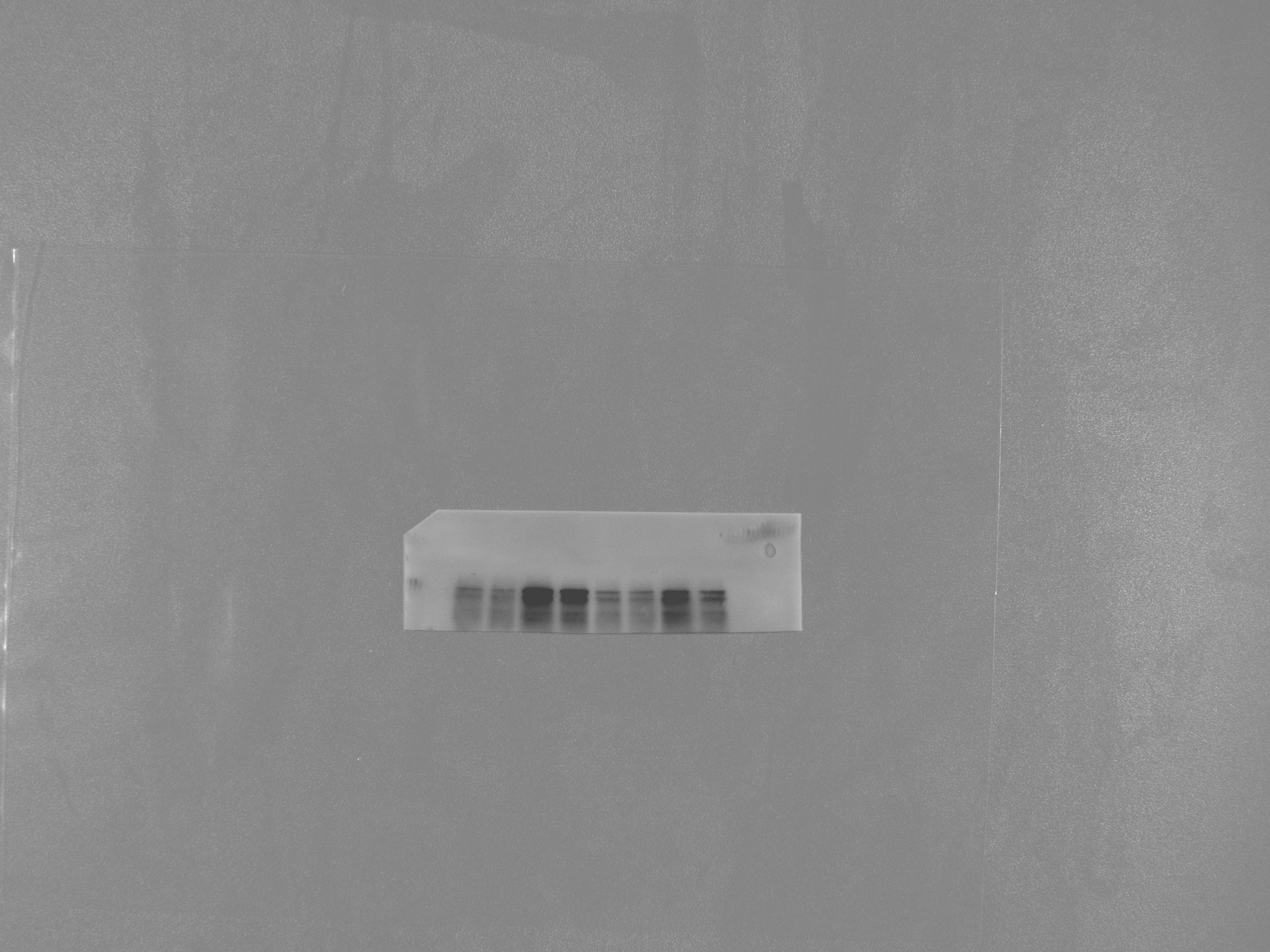

Supplement: S2 File — Original pictures of the western blot analysis in the manuscript. (ZIP) [file pone.0295432.s002.zip › Western blot results/Fig 6 B-Nav18-1.tif]

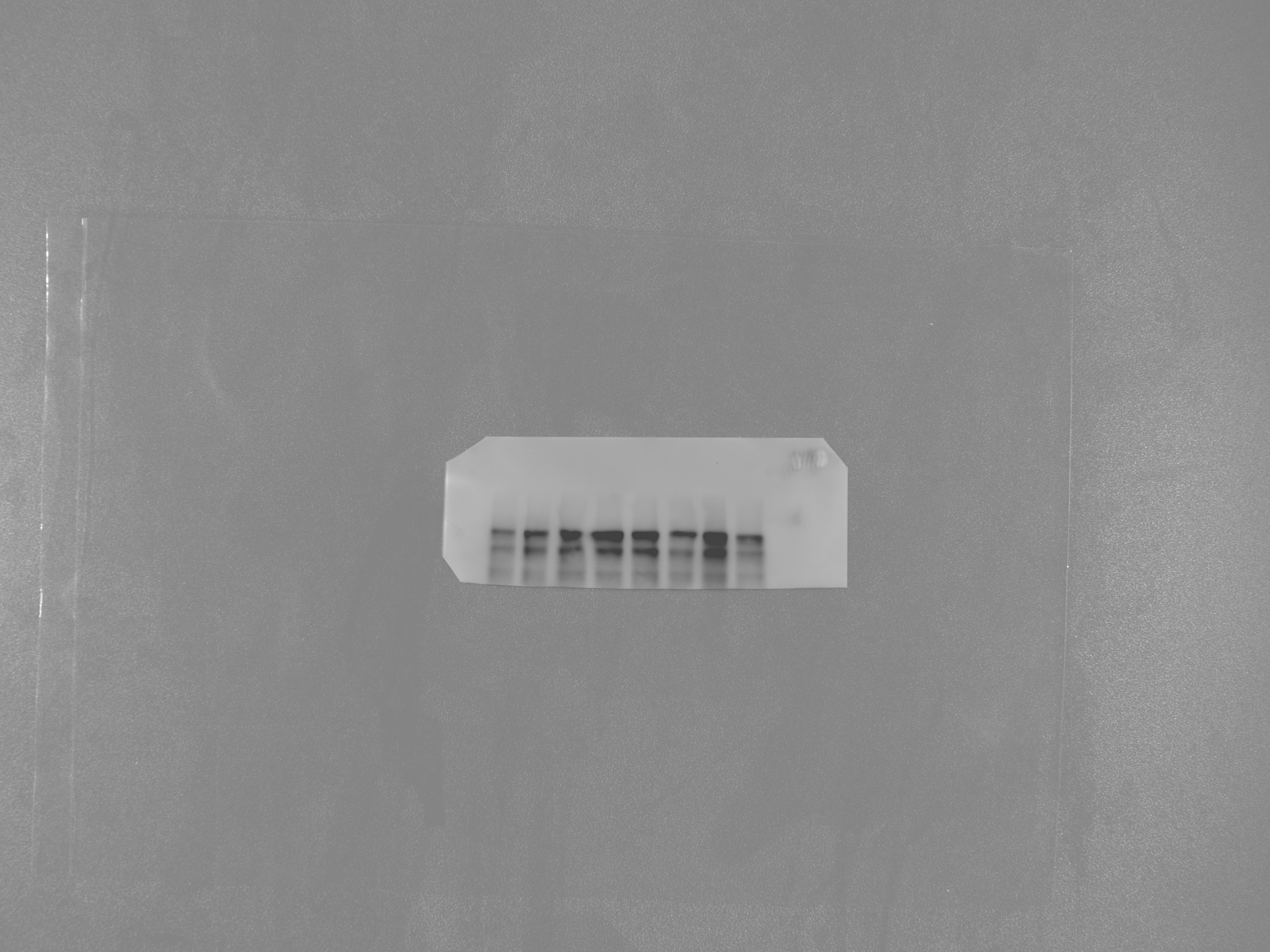

Supplement: S2 File — Original pictures of the western blot analysis in the manuscript. (ZIP) [file pone.0295432.s002.zip › Western blot results/Fig 6 B-Nav18-2.tif]

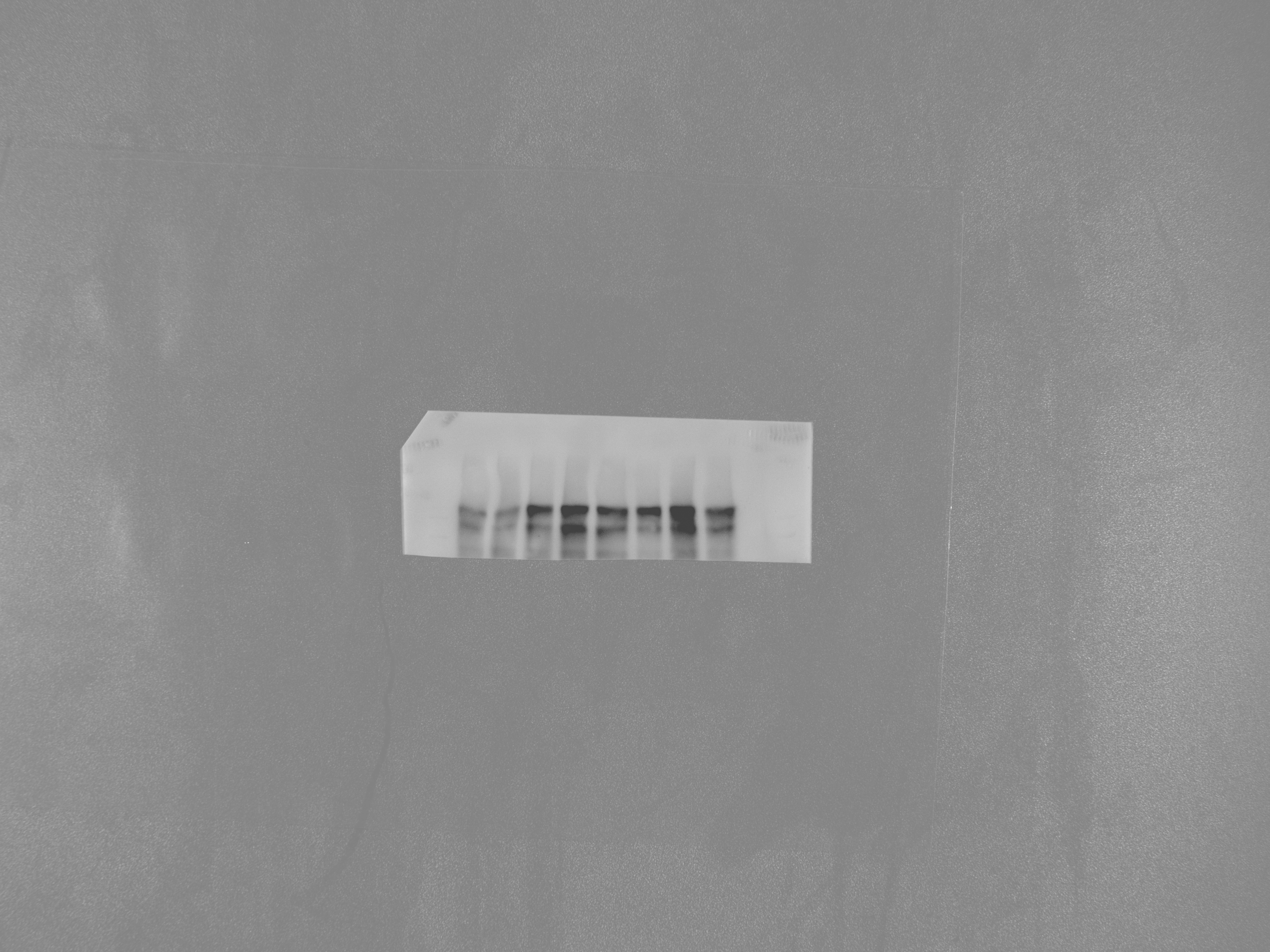

Supplement: S2 File — Original pictures of the western blot analysis in the manuscript. (ZIP) [file pone.0295432.s002.zip › Western blot results/Fig 6 B-Nav18-3.tif]

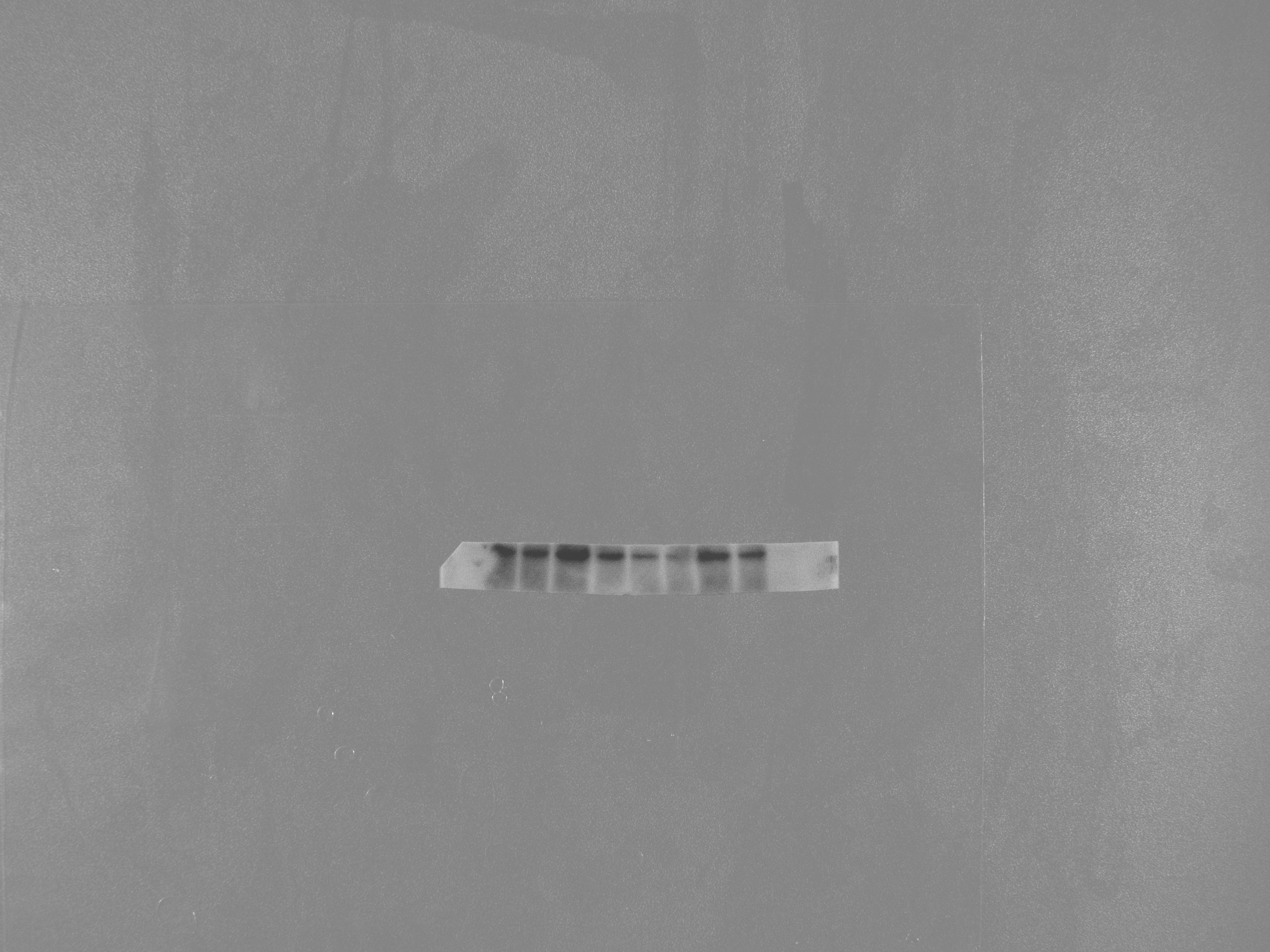

Supplement: S2 File — Original pictures of the western blot analysis in the manuscript. (ZIP) [file pone.0295432.s002.zip › Western blot results/Fig 6 C-cox2-1.tif]

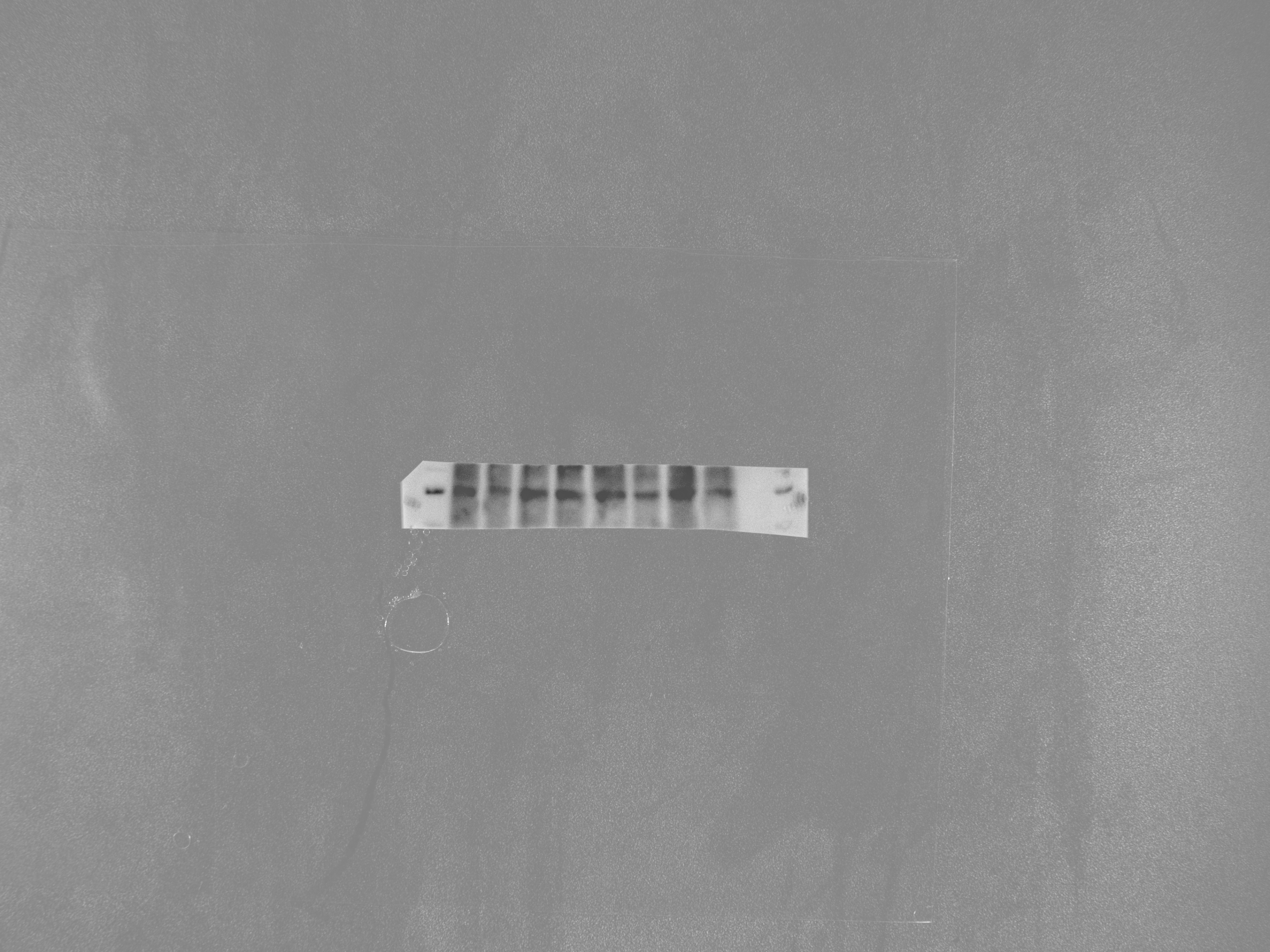

Supplement: S2 File — Original pictures of the western blot analysis in the manuscript. (ZIP) [file pone.0295432.s002.zip › Western blot results/Fig 6 C-cox2-2.tif]

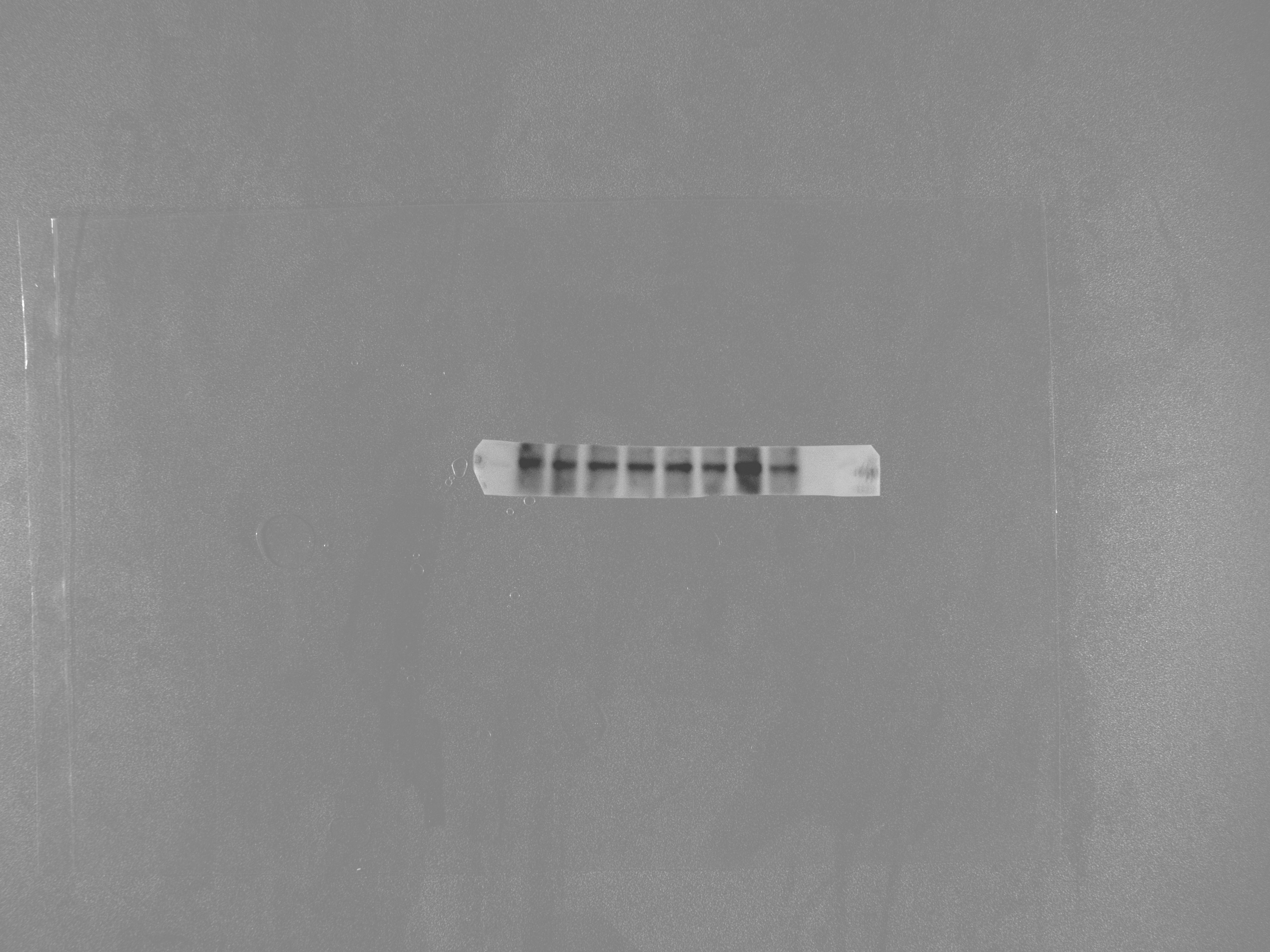

Supplement: S2 File — Original pictures of the western blot analysis in the manuscript. (ZIP) [file pone.0295432.s002.zip › Western blot results/Fig 6 C-cox2-3.tif]

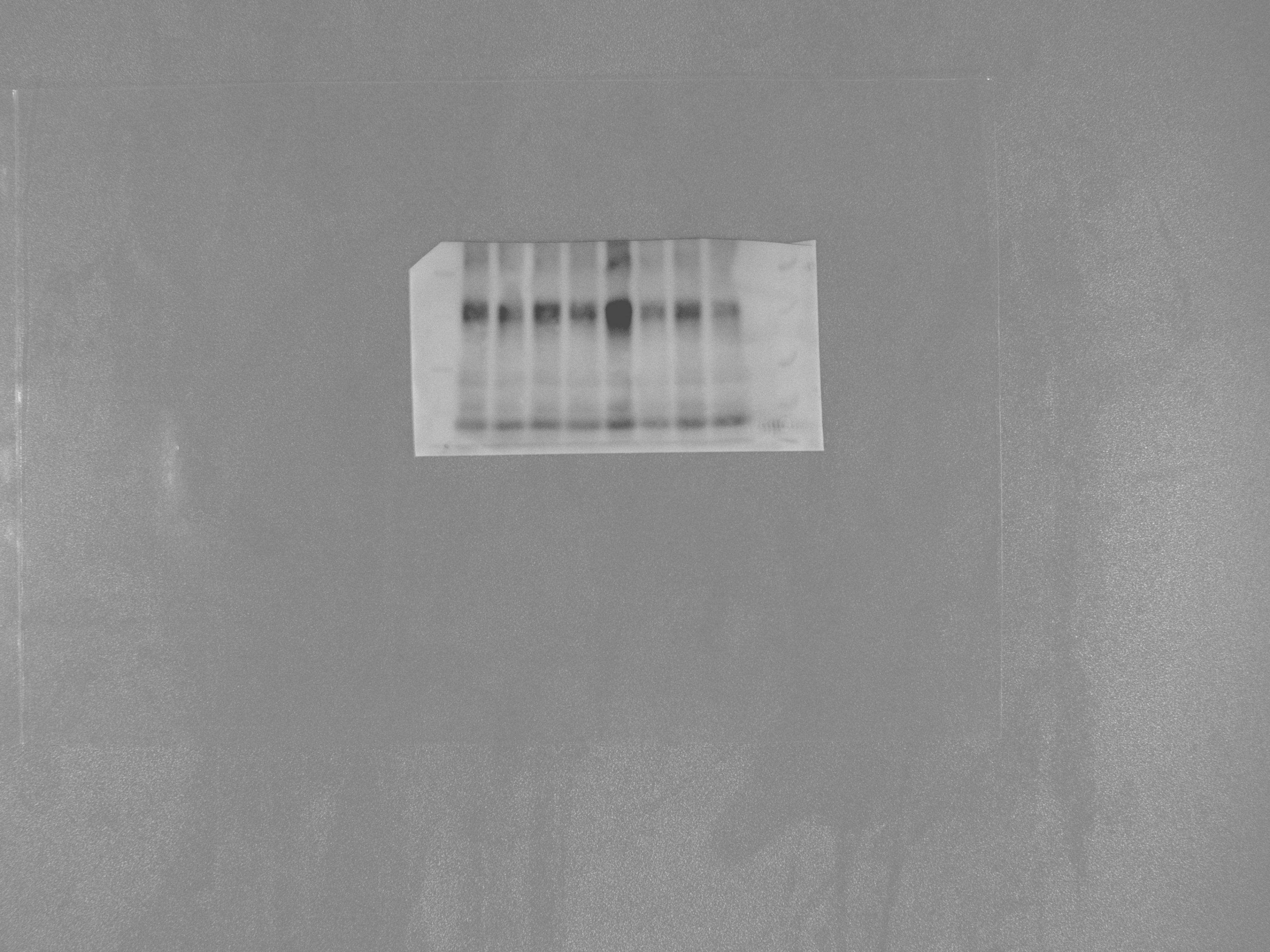

Supplement: S2 File — Original pictures of the western blot analysis in the manuscript. (ZIP) [file pone.0295432.s002.zip › Western blot results/Fig 6 D-cb1-1.tif]

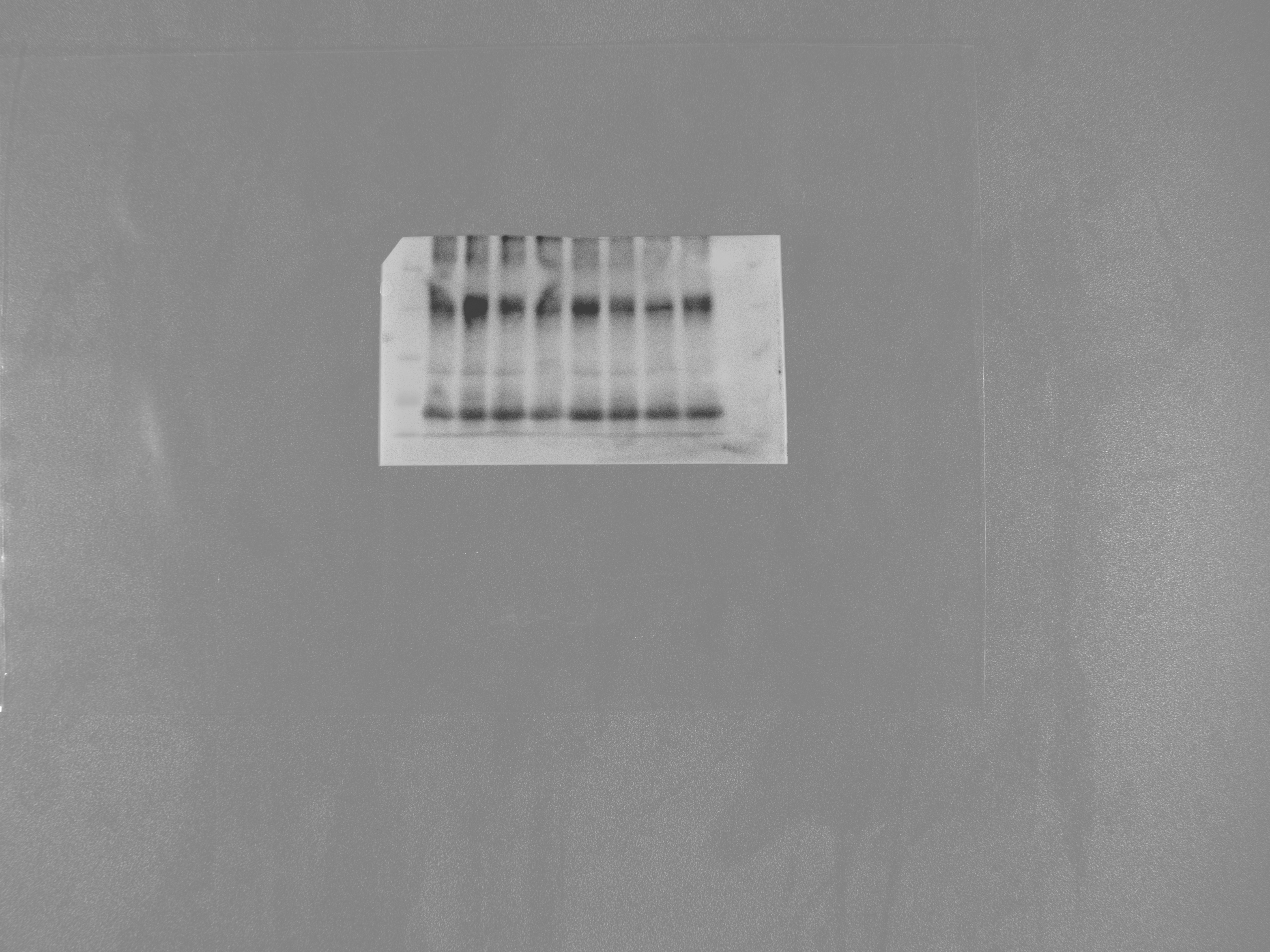

Supplement: S2 File — Original pictures of the western blot analysis in the manuscript. (ZIP) [file pone.0295432.s002.zip › Western blot results/Fig 6 D-cb1-2.tif]

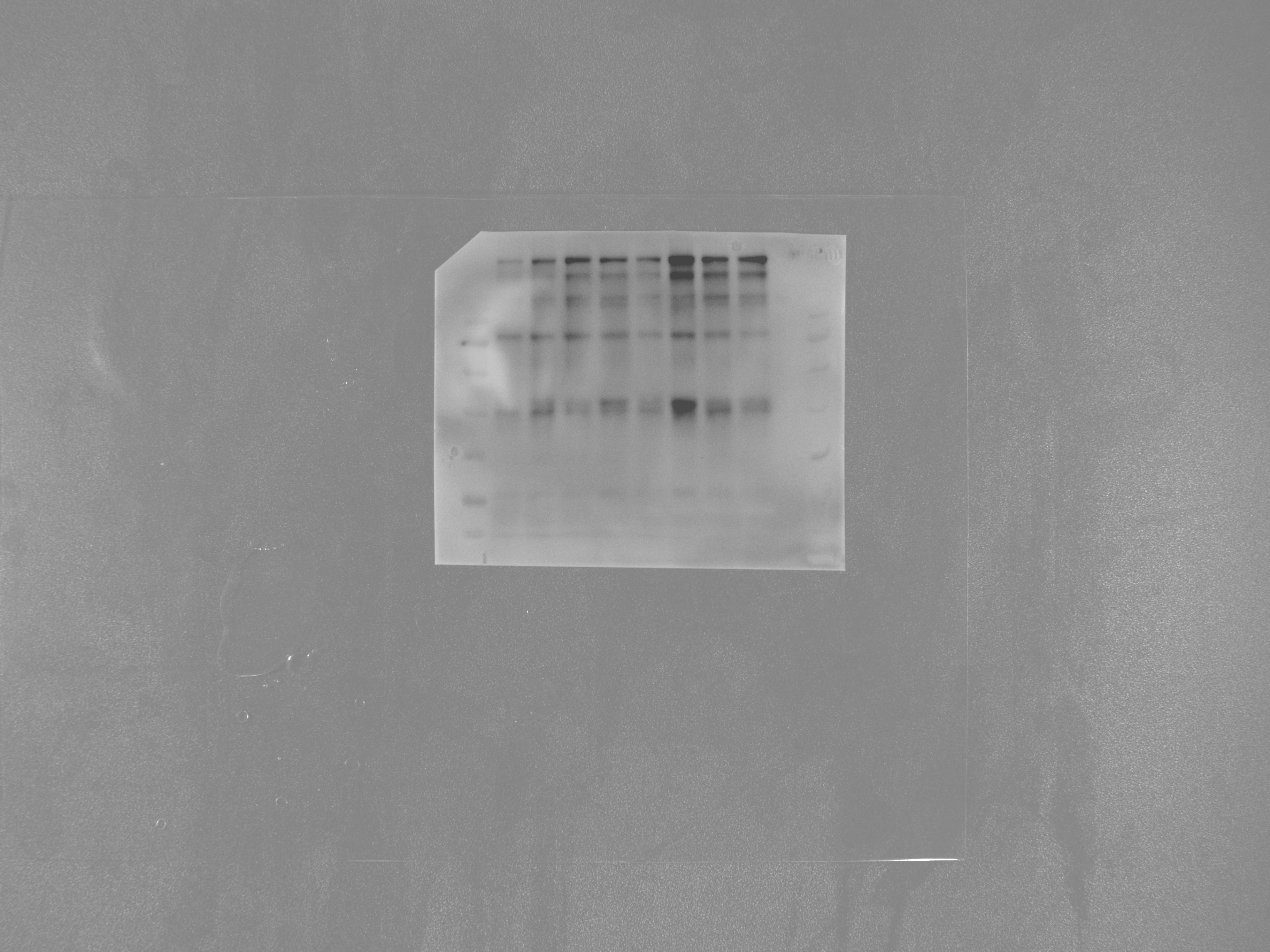

Supplement: S2 File — Original pictures of the western blot analysis in the manuscript. (ZIP) [file pone.0295432.s002.zip › Western blot results/Fig 6 D-cb1-3.tif]

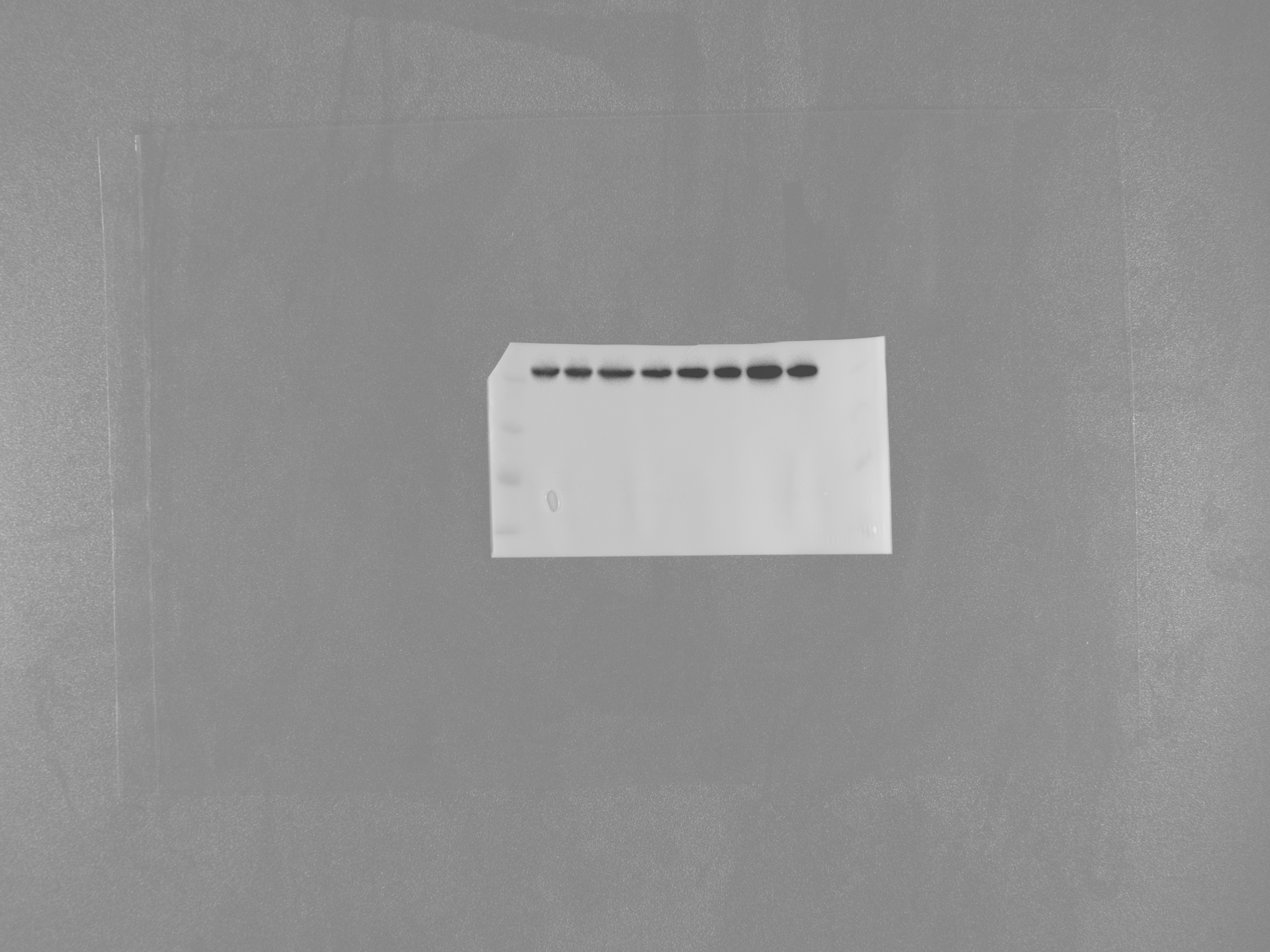

Supplement: S2 File — Original pictures of the western blot analysis in the manuscript. (ZIP) [file pone.0295432.s002.zip › Western blot results/Fig 6-tubulin-1.tif]

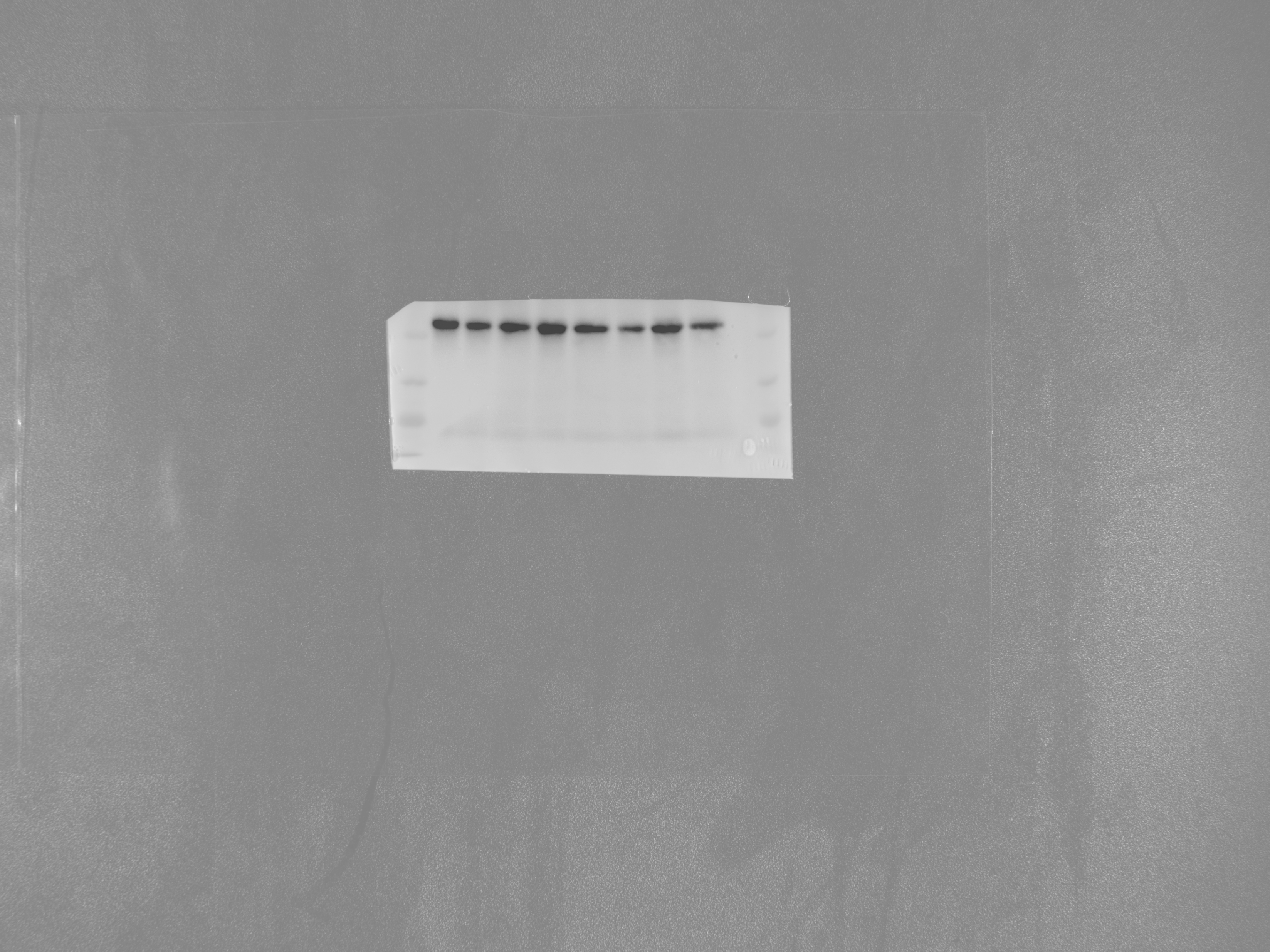

Supplement: S2 File — Original pictures of the western blot analysis in the manuscript. (ZIP) [file pone.0295432.s002.zip › Western blot results/Fig 6-tubulin-2.tif]

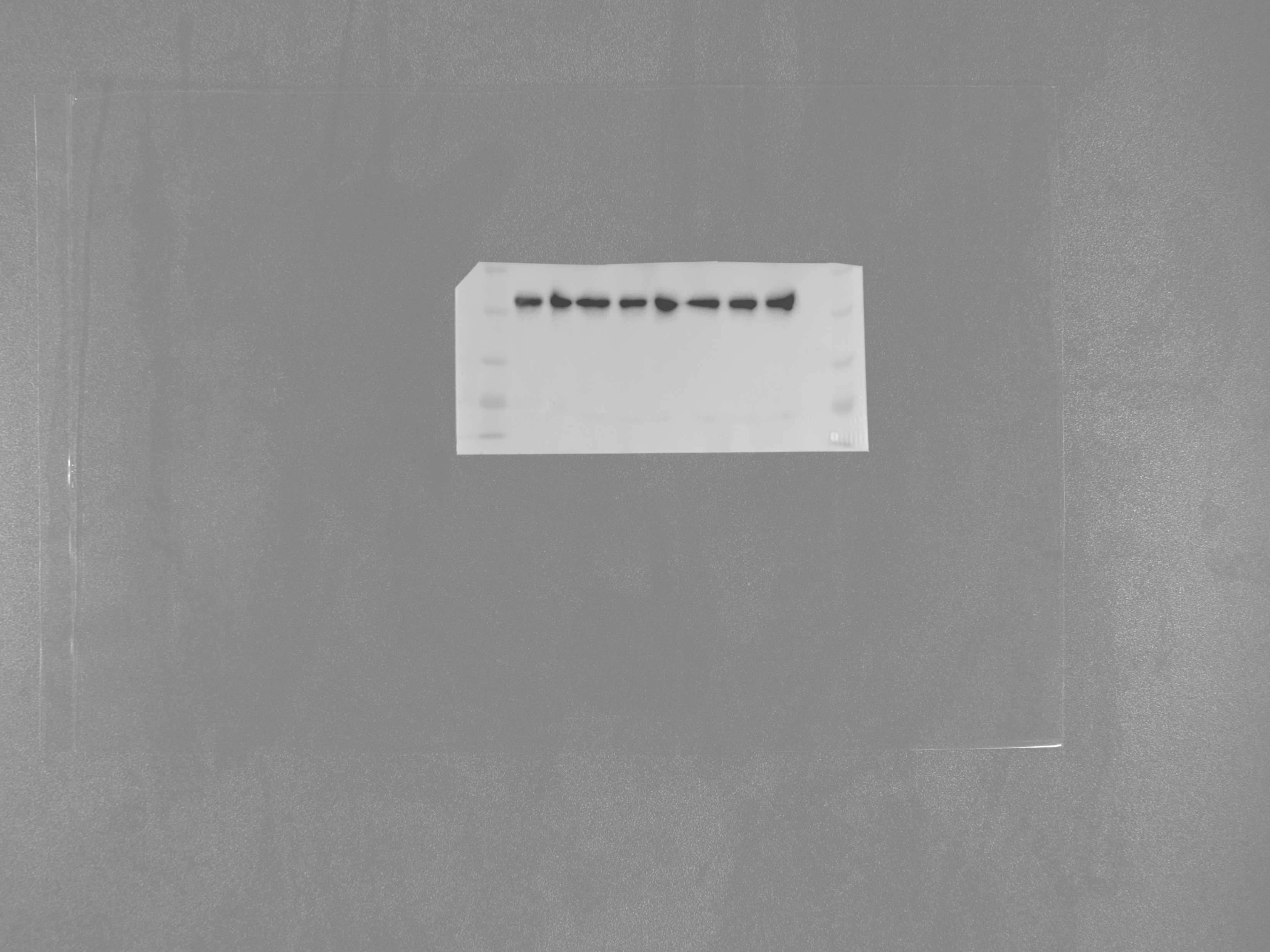

Supplement: S2 File — Original pictures of the western blot analysis in the manuscript. (ZIP) [file pone.0295432.s002.zip › Western blot results/Fig 6-tubulin-3.tif]
